# Three Neglected STARD Criteria Reduce the Uncertainty of the Liver Fibrosis Biomarker FibroTest-T2D in Metabolic Dysfunction-Associated Steatotic Liver Disease (MASLD)

**DOI:** 10.3390/diagnostics15101253

**Published:** 2025-05-15

**Authors:** Thierry Poynard, Olivier Deckmyn, Raluca Pais, Judith Aron-Wisnewsky, Valentina Peta, Pierre Bedossa, Frederic Charlotte, Maharajah Ponnaiah, Jean-Michel Siksik, Laurent Genser, Karine Clement, Gilles Leanour, Dominique Valla

**Affiliations:** 1 Medical Faculty Pitié Salpêtrière, Sorbonne University, 75005 Paris, France; raluca.pais@aphp.fr (R.P.); judith.aron-wisnewsky@inserm.fr (J.A.-W.); frederic.charlotte@aphp.fr (F.C.); laurent.genser@aphp.fr (L.G.); karine.clement@inserm.fr (K.C.); 2 BioPredictive, 75007 Paris, France; olivier.deckmyn@biopredictive.com (O.D.); valentina.peta@biopredictive.com (V.P.); 3 Assistance Publique Hôpitaux de Paris, Hôpital Pitié-Salpêtrière, 75013 Paris, France; jean-michel.siksik@aphp.fr; 4 Institut National de la Santé et de la Recherche Médicale, 75013 Paris, France; maharajah.ponnaiah@inserm.fr; 5 Nutrition Department, Assistance Publique Hôpitaux de Paris, Pitié-Salpêtrière Hospital, 75013 Paris, France; 6 Centre de Recherche Saint Antoine, INSERM UMRS_938, 75012 Paris, France; 7 UMR1149 (CRI), Inserm, Université Paris Cité, 75018 Paris, France; pierre.bedossa@inserm.fr (P.B.);; 8 Liverpat, 75116 Paris, France; 9 CNRS UMR8507, Laboratoire Génie Électrique et Électronique de Paris (GeePs), Sorbonne Université, 75252 Paris, France; gilles.leanour@sorbonne.fr; 10 Service d’Hépatologie, AP-HP, Hôpital Beaujon, 92110 Clichy-la-Garenne, France

**Keywords:** uncertainty, Obuchowski measure, early liver fibrosis, granularity, UK BioBank, biopsy length

## Abstract

**Background/Objectives:** Bariatric surgery (BS), drugs approved for type-2-diabetes (T2D), obesity, and liver fibrosis (resmetirom) announce the widespread use of fibrosis tests in patients with metabolic liver disease (MASLD). An unmet need is to reduce the uncertainty of biomarkers for the diagnosis of the early stage of clinically significant fibrosis (eF). This can be achieved if three essential but neglected STARD methods (3M) are used, which have a more sensitive histological score than the standard comparator (five-tiers), the weighted area under the characteristic curve (wAUROC) instead of the binary AUROC, and biopsy length. We applied 3M to FibroTest-T2D to demonstrate this reduction of uncertainty and constructed proxies predicting eF in large populations. **Methods:** For uncertainty, seven subsets were analyzed, four included biopsies (*n* = 1903), and to assess eF incidence, three MASLD-populations (*n* = 299,098). FibroTest-T2D classification rates after BS and in outpatients-T2D (*n* = 402) were compared with and without 3M. In MASLD, trajectories of proxies and incidence against confounding factors used hazard ratios. **Results:** After BS (110 biopsies), reversal of eF was observed in 16/29 patients (84%) using seven-tier scores vs. 3/20 patients (47%) using five-tier scores (*p* = 0.005). When the biopsy length was above the median, FibroTest-T2D wAUROC was 0.90 (SD = 0.01), and the wAUROC was 0.88 (SD = 0.1) when the length was below the median (*p* < 0.001). For the first time, obesity was associated with eF before T2D (*p* < 0.001), and perimenopausal age with apoA1 and haptoglobin increases (*p* < 0.0001). **Conclusions:** Validations of circulating biomarkers need to assess their uncertainty. FibroTest-T2D predicts fibrosis regression after BS. Applying 3M and adjustments could avoid misinterpretations in MASLD surveillance.

## 1. Introduction

The prevalence of metabolic dysfunction-associated steatotic liver disease (MASLD) is increasing, making it a leading cause of liver fibrosis progression, cirrhosis, and liver cancer [1]. No treatments have yet been validated for cirrhosis, but effective treatments are available for non-cirrhotic cases: weight loss and lifestyle changes, bariatric surgery (BS), and resmetirom [2,3,4,5,6,7]. Therefore, a major unmet need is the validation of circulating noninvasive tests (NITs) that can be measured to diagnose fibrosis before cirrhosis develops [8].

In the context of statistical diagnostic methods, accuracy is defined as the percentage of patients correctly classified as true positive or true negative and used when validating NITs for fibrosis.

The accuracy of these NITs refers to the degree of certainty in a given measurement or outcome. In the context of statistical diagnostic methods, accuracy is defined as the percentage of patients correctly classified as true positive or true negative and used when validating NITs for fibrosis. In the clinical real life, accuracy is “the closeness of the agreement between the result of a measurement and a true value of the thing being measured”; accuracy is a qualitative concept expressed as being high or low, but not with numbers. Without a perfect comparator with appropriate granularity, even with a perfect fibrosis NIT and ideal biopsies, a 90% correct classification cannot be achieved, and this figure decreases to 80% with biopsies smaller than 20 mm [9,10,11,12,13,14,15].

Therefore, any comparison between NITs must account for their comparator uncertainty, the risk–benefit ratio, the major confounding factors (CFs), and the context of use. Three rarely applied but essential statistical diagnostic methods (3M) should accelerate the approval of NITs; the first method is the choice of the comparator, as recently illustrated by the resmetirom trial [7]. For the first time, 3M appeared briefly in the Standards for Reporting Diagnostic Accuracy (STARD) statement in criterion #15-2015-version: “How indeterminate index test or reference standard results [comparators] were handled” [16,17].

The uncertainty of biopsy (the comparator) is highly associated with (1) the cutoffs defining each fibrosis stage and the number of tiers (granularity); (2) the choice of the statistical method, which is the weighted area under the curve (wAUROC or Obuchowski measure) [16,17,18,19]; and (3) the biopsy length [10,11,12,13,14]. wAUROC is more suitable than standard bAUROC because it gives a single estimate of the overall accuracy, which is the mean of all possible binary AUROC combinations whereas bAUROC is depending of each stage prevalence (spectrum effect) [19]. 

If there are three stages for fibrosis (F0-F1-F2), wAUROC includes the mean of (F0 vs. F1; F0 vs. F2, and F1 vs. F2). If there is a regular spectrum (33% F0, 33% F1, 33% F2), the accuracy of wAUROC and all binary AUROCs could be interpretable, but if the spectrum is 1% F0, 33% F1 and 66% F2, the accuracy of binary-F2 vs. F0 will be completely biased by this spectrum effect.

It is commonly assumed that a small amount of uncertainty (less than 3%) in the comparator’s classifications negligibly affects the performance of a diagnostic test [9]. This is not true for fibrosis NITs, for which the uncertainty is above 20% [10,11,12,13,14]. An extension of STARD for reporting on liver fibrosis tests (Liver-FibroSTARD) recommends methods in criterion #13.7, specifically “the methods useful for the control of the spectrum effect, such as the Obuchowski method and DANA score” [16,17,18,19].

Here, we postulate that the most cited available NITs should be revisited according to the 3M. In the resmetirom landmark study, a more sensitive comparator was defined using the three substages of the F1 stage of the standard clinical research network scoring system named CRN [7,20]. The granularity of the standard CRN score becomes more sensitive when modified in a CRN-F1B score: F1B is defined as early fibrosis (eF), the other non-cirrhotic clinically significant stages being the CRN standards F2, F3, and F4. The revised stage F0 includes the biopsy without fibrosis and the substages F1A and F1C, which are considered non-clinically significant fibrosis [7].

The most cited NITs, such as the FibroSure/FibroTest and the Enhanced Liver Fibrosis score (ELF) for MASLD [8], have been recommended worldwide in viral hepatitis and alcoholic liver disease without ideal trials. A simple NIT, the fibrosis-4-index (FIB4), is less expensive than patented NITs, but it has lower performance for eF; in a prospective cohort of 5715 patients with sustained hepatitis-C virological response, the prevalence of severe liver fibrosis decreased from only 26% to 17% after 4 years [21].

Before the approval of resmetirom, a systematic review and meta-analysis reported pharmacologic efficacy against fibrosis for five drugs based on NITs, including FibroSure/FibroTest and ELF. ELF score improved after resmetirom treatment [7], and FibroSure/FibroTest results improved after obeticholic acid treatment, both relative to placebo [22].

We present two post hoc proofs of concept (Table 1). The first compared the NIT performance for the diagnosis of eF (the main endpoint) in patients with biopsies when 3M were applied versus when they were not. The improvement in performance allowed for the construction of proxy-NITs, which were applied in large populations. This second concept allowed the construction of trajectories of eF stages, steatosis, and inflammation stages to be compared according to sex, T2D, and obesity for the first time.

## 2. Patients and Methods

### 2.1. Ethics

All authors had access to the data and reviewed and approved the final manuscript. This retrospective study was performed in accordance with the Declaration of Helsinki, and the details are available in previous publications (Table 1). The pre-analytical and analytical NIT procedures were recommended by BioPredictive, including the exclusion criteria and the use of medical security control algorithms to assess non-reliable results [27]. Details are provided in Supplementary File S1. All data were analyzed anonymously.

### 2.2. Patients

The following four cohorts included patients with biopsies: (1) the prospective BARiatric study of the foundation for Innovation in CArdiometabolism and Nutrition (BARICAN) cohort, including 55 patients before and after BS [5], as summarized in Supplementary File S1 and Figure S2; (2) the prospective QuidNash consortium (https://rhu-quidnash.com/about-the-project/, accessed on 4 May 2025), including 402 patients with T2D [14,24]; (3) the Liver Injury in Diabetes and Obesity (LIDO) study, including 51 patients with MASLD who received two biopsies on the same day [13]; and (4) the retrospective Fibrosis-TAGS (Truth in the Absence of a Gold Standard) study, including 1293 biopsies, with large surgery biopsies as a nearly perfect comparator [11].

The next three cohorts included patients at risk of MASLD without biopsies, with fibrosis stages, steatosis, and inflammation grades estimated using the proxies: (5) the prospective UK Biobank cohort, including 159,794 middle-aged, apparently healthy participants [24] (inclusion details provided in Supplementary Figure S2; characteristics according to sex, BS history, and menopause are provided in Supplementary Tables S1–S3); (6) the France FibroTest database, including 67,278 patients [25]; and (7) the US FibroTest database, including 72,026 patients [26]. The four CFs were assessed in all these patients (Table 1 and Supplementary Table S4).

The main characteristics—specifically age (57 years old), percentage of women (53%), and body mass index (BMI) (31 kg/m^2^)—were similar in the UK Biobank and the US FibroTest cohorts. The prevalence of T2D in the UK Biobank was only 6%, as this cohort excluded participants not healthy during recruitment; this prevalence was much lower than that in the France FibroTest (16%) and US FibroTest participants (22%). The France FibroTest cohort had a much lower percentage of females (41%), and participants had a lower average BMI (28 kg/m^2^), in comparison with the other subsets.

### 2.3. Methods

#### 2.3.1. First Aim: To Compare Two Scoring Systems, Both with 5-Tiers, eF Being More Sensitive than the Standard CRN

In the longitudinal BARICAN cohort [6], we assessed the post hoc performance of FibroTest-T2D to identify patients with eF regression after BS, as summarized in Supplementary File S2, and to exclude patients (Supplementary Table S5).

In the Fibrosis-TAGS study, using large surgical biopsies as the true reference (gold standard comparator = fibrosis area), we conducted post hoc comparisons for the first time to determine the uncertainty of three possible proxy comparators: a biopsy proxy in MASLD using the CRN; a circulating proxy using a FibroTest proxy; and an imaging proxy using a VCTE proxy. Because the F1 substages were not assessed, we constructed a uniform scoring score (seven-tiers) that uses the normalized area of stages F0 to F6 divided by seven [11].

#### 2.3.2. Second Aim: To Assess the Performance of FT-T2D Using wAUROC or the Adjusted Binary AUROC Instead of the Standard Binary AUROC (bAUROC)

To compare different spectra without making direct comparisons, it is mandatory to use the wAUROC [11,16,17,18,19] (Supplementary File S4). Because few studies have used the wAUROC, here, we systematically applied an index of fibrosis spectrum variability called DANA (Difference between Advanced and Non-Advanced fibrosis) in patients at risk of MASLD to predict the adjusted bAUROC for the 5-tier CRN stages [17,19,28].

#### 2.3.3. Third Aim: To Assess the Impact of Biopsy Sample Length

Doubling the length of the median biopsy from 20 mm to 40 mm increased the prevalence of bridging fibrosis (stage F3) using CRN from 25% to 33%, and it reduced the misclassification rate to 8% in MASLD [13]. We previously used the published comparator of the misclassification rate [9,14], and using a true reference with large surgical biopsies, we assessed the biopsy uncertainty [11], as detailed in Supplementary File S4. With a 17 mm median biopsy specimen, the maximum expected bAUROC for an ideal marker decreased to 0.70 because of the 30% misclassification rate of the biopsy. Here, we stratified the wAUROCs using the median biopsy lengths as cutoffs (Table 2).

#### 2.3.4. Fourth Aim: To Assess the Trajectories of Fibrosis, Inflammation, and Steatosis Stratified by Sex, T2D, and Obesity

We built proxies of FibroTest-T2D (FT-2tD-proxy), SteatoTest-T2D (ST-t2D-proxy), and NashTest-T2D (NT-t2D-proxy) that were independent of the age of the participants and used separately in women and men. This construction permitted us to avoid co-linearity and assess the fibrosis progression rate (FPR) from birth to the first occurrence of eF by sex. First, in the QuidNash cohort, we performed a multiple logistic regression using the components of FibroTest-(FibroSure-Plus in the US) that predict the stage eF, the comparator endpoint in the 402 consecutive patients with T2D. Second, we used the Bland–Altman plots and limits of agreement (BA-LOA) to assess the significance of linking with the original and proxies stratified by country (USA, France) and sex. As previously described [28], the final step was to assess the FPR using the cumulative hazard ratio from birth to the first occurrence of the stage of interest, in this case eF, in the large US and French cohorts according to CFs and the earlier features of steatosis and inflammatory grades using similar proxy constructions. The variability of five components (alpha-2-macroglobulin [A2M], apolipoprotein A1 [apoA1], haptoglobin, gamma-glutamyl transpeptidase [GGT], and bilirubin) was assessed in large populations at risk of MASLD. In the UK Biobank, we focused on the postmenopausal rise in the rate of MASLD.

### 2.4. Sensitivity Analyses

Patients underwent routine FibroTest-T2D assessments, which were performed before and after the BS protocol to increase the FPR assessments; specifically, tests were conducted before BS (between the preparation routine and biopsy 1), between BS and follow-up (biopsy-2), and between biopsy-2 and the latest routine FibroTest-T2D.

## 3. Results

### 3.1. First Aim: Advantage of a More Sensitive (eF) Histological Comparator

**Figure 1:**
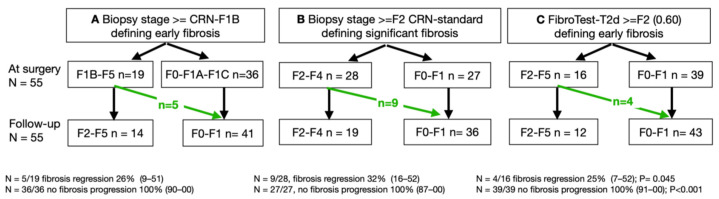
Bariatric surgery performance for reducing fibrosis. (**A**) CRN-F1B was used as described in the resmetirom trial [7]. (**B**) Standard CRN was used [20]. (**C**) The FibroTest-T2D blood test was used [14,23]. The revised stage F0 includes no-fibrosis and the very low fibrosis substages F1, F1A, and F1C [7]. The three methods observed the absence of 100% (95% CI 91%-1; *p* < 0.001) of fibrosis progression.

In the BARICAN study, 19 of 55 patients (35%; 95% CI 22–49) were classified as stage eF or higher at the time of surgery, and only 14 patients (25%; 95% CI 15–39) were classified as such 6 years later (F0/F1A/F1C; Figure 1A), which was a significant decrease (Nam RMLE-score = 9.7; *p* = 0.002).

Using CRN, 28 of 55 patients (51%; 95% CI 37–65) were classified as having a significant fibrosis stage of at least F2 (F2/F3/F4; Figure 1B), which decreased to 19 patients (35%; 95% CI 22–47) without significant fibrosis (F0/F1), a non-significant decrease (Nam RMLE Score = 1.4; *p* = 0.24).

When the FT-T2D was used, 16 of 55 patients (25%; 95% CI 15–39) had score of at least 0.60, the cutoff chosen for eF, which decreased to 12 patients (22%; 95% CI 12–35) without significant fibrosis (FT-T2D < 0.60, Figure 1C), a highly significant decrease (Nam RMLE-Score = 14.3; *p* = 0.0002).

**Figure 2:**
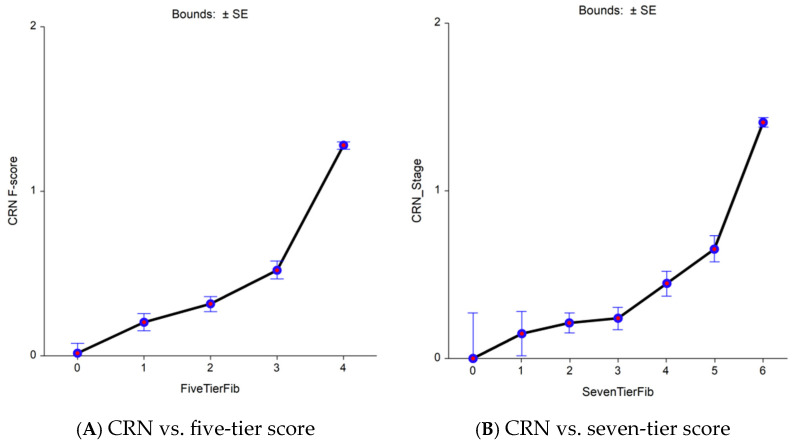

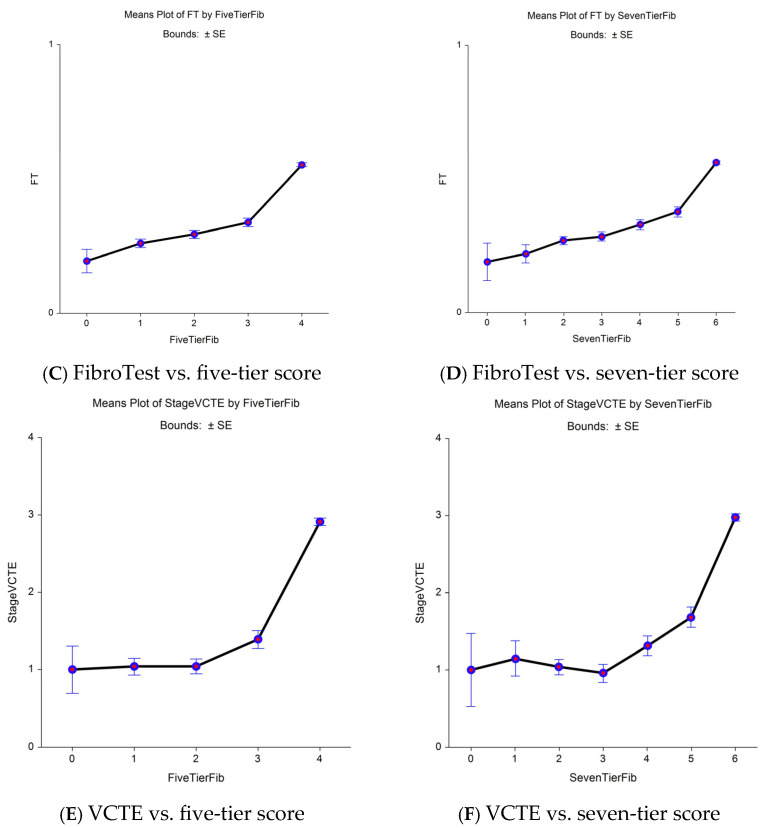
CRN, FibroTest, and VCTE (*y*-axis) vs. area of fibrosis (*x*-axis) as the comparator reference (*n* = 1726). Increasing the granularity of the reference improved comparisons between non-invasive tests. (**A**) Biopsy CRN—five-tier stages vs. five-tier fibrosis area. (**B**) Biopsy CRN—seven-tier stages vs. seven-tier fibrosis area. (**C**) FibroTest—five-tier stages vs. five-tier fibrosis area. (**D**) FibroTest—seven-tier stages vs. seven-tier fibrosis area. (**E**) Seven-tier VCTE stages vs. five-tier fibrosis area. (**F**) Seven-tier VCTE stages vs. seven-tier fibrosis area.

In the Fibrosis-TAGS cohort, the nearly perfect comparator (fibrosis area) permitted a decrease in the uncertainty of FibroTest in comparison with VCTE for the diagnosis of early bridging revealed by sensitive seven-tier vs. standard five-tier. A total of 2160 virtual biopsies were available and scored using CRN as F0 (*n* = 1080; 50%), F1 (*n* = 540; 25%), and F2 (*n* = 540; 25%) (Figure 2 and Supplementary Table S6). The analyses of the CRN, FibroTest, and VCTE contemporaneous values revealed a significant association between the CRN and FibroTest values, which was linear in the early bridging zone (F2–F3) when the reference used CRN (Figure 2A,2B). Two slopes were observed between F0 and F1 when the seven-tier score was used (Figure 2C) and between the F1 and F2 fibrosis categories for FibroTest (Figure 2D). For VCTE, no increase in stiffness was observed between the early fibrosis stages both when the five-tier score (Figure 2E) or the seven-tier (Figure 2F) scores, suggesting false negative cases.

### 3.2. Second Aim: Performance of FT-T2D vs. FibroTest Using wAUROC or bAUROC

FibroTest-T2D had a significantly higher (*p* < 0.001) wAUROC (median [SD]; 0.86 [0.01]) than the regular FibroTest (0.80 [0.01]), both in 402 patients with T2D (QuidNash) [14,23] and 55 patients with BS (BARICAN) and 110 paired biopsies [6], regardless of the fibrosis scoring system (Table 2).

### 3.3. Third Aim: Impact of the Biopsy Sample Length, a Major Factor of Uncertainty

In the QUIDNASH cohort, when the biopsy length was above the median, the FibroTest-T2D wAUROC results were significantly higher than those of the regular FibroTest when the biopsy length was above the median: 0.85 vs. 0.80 (*p* = 0.002) respectively; by comparison, when the length was below the median, it was 0.86 vs. 0.84 (*p* = 0.12), respectively (Table 2).

### 3.4. Fourth Aim: Variability of Early Fibrosis Trajectories in Large Populations

The FT-T2D proxy had a significant bAUROC (0.77; 95% CI 0.72–0.81; *p* < 0.001) for the diagnosis of eF using regression analysis, similar to that of the FibroTest-T2D (bAUROC = 0.77; 95% CI 0.72–0.82; *p* = 0.84). BA-LOA among patients with NITs and biopsies is detailed in Supplementary Figure S3. Correlation coefficients were highly significant (*p* < 0.001) and varied from 0.82 to 0.94: NITs-T2D-proxy-women (*n* = 159)/men (*n* = 243) = 0.94/0.88; NT-T2D = 0.80/0.80; ST-T2D = 0.85/0.82. The bias and LOA were not perfect and varied as follows: 0.03 for steatosis, 0.25 for fibrosis, and 0.27 for Nash.

**Figure 3:**
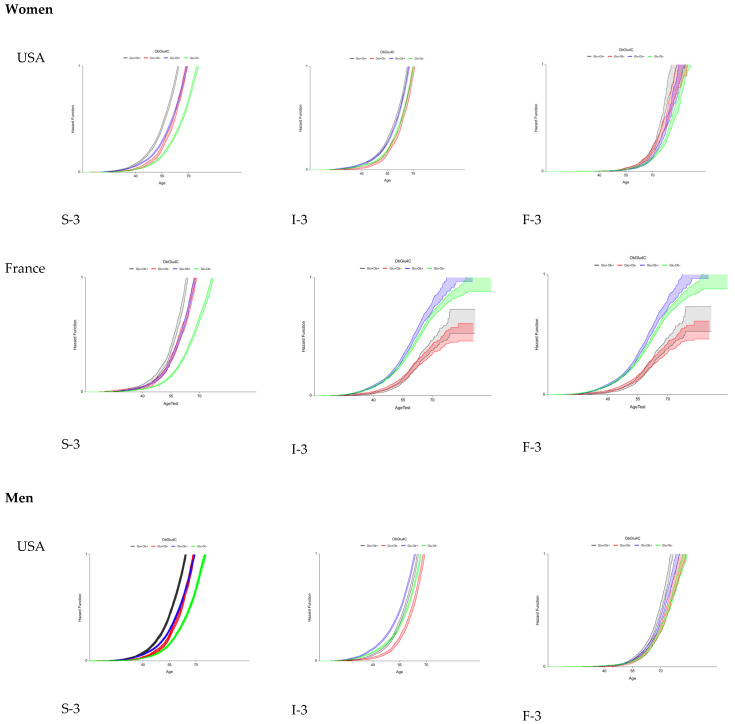

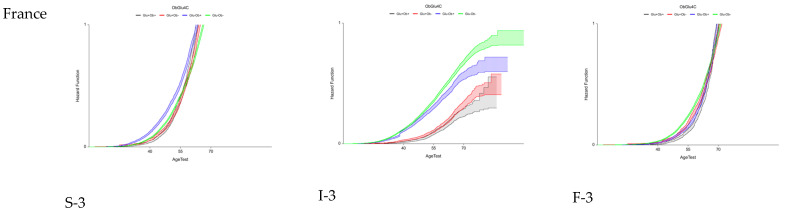
Fibrosis progression rates (FPRs) to early fibrosis in US and French populations at risk of MASLD.

Using these proxies, the FPR of eF was assessed for the first time in French and US cohorts of patients at risk of MASLD, stratified by country, sex, T2D, and obesity, and simultaneously with the trajectories of the two earlier features: steatosis and inflammation (Figure 3).

For eF occurrence, the trajectories were similar regardless of the country and sex. Surprisingly, obesity without T2D was the pre-existing risk factor associated with eF occurrence. T2D and obesity were the pre-existing factors associated with the occurrence of severe steatosis and inflammation.

Regarding trajectories, in the UK Biobank subset, apoA1 increased until 50 years of age in women regardless of BMI (Supplementary Figure S4A). In women with T2D who were not overweight, apoA1 increased at perimenopausal age (Supplementary Figure S4A), as confirmed in the subset with NMR (Supplementary Figure S4C; Supplementary Tables S6 and S8). In men with T2D and women with a BMI ≥ 27, the apoA1 increase completely disappeared (Supplementary Figure S4A–C,E. More details are provided for the UK Biobank participants with a history of BS (*n* = 681) (Supplementary Table S2).

In the US and French subsets, haptoglobin (Supplementary Figure S5A), and A2M (Supplementary Figure S6) were associated with age.

### 3.5. Sensitivity Analyses (Supplementary File S7)

At the surgery time, the diagnosis of MASH grades A2/A3 using NashTest-T2D was significant, with a bAUROC (IQR; *p*-value) = 0.68 (0.51–0.80; *p* = 0.007), which was higher than that using AST (0.52; 0.34–0.67; *p* = 0.02) (Supplementary Figure S7A); and at the second biopsy, with a bAUROC of 0.77 (0.61–0.87; *p* < 0.001), but this did not differ from AST (0.71; 0.51–0.83; *p* = 0.35) (Supplementary Figure S7B).

Because steatosis was present in all cases at the time of surgery, the assessment of SteatoTest-T2D performance was possible only at the time of the second biopsy. For the diagnosis of grades S2 and S3 (prevalence = 0.15), the difference was significant (*p* = 0.001), with an AUROC of 0.71 (0.49–0.84; *p* = 0.01), but it was not significant using triglycerides (*p* = 0.95) (Supplementary Figure S3C). The FPR decrease for FibroTest-T2D between biopsies was significant only in men (median [IQR]: −2.6% [−3.6% to 1.2%], *p* = 0.02). 

## 4. Discussion

The limitations and strengths of our results were compared with recently published NIT reviews [2,8,15,22,29]. These reviews achieved a consensus on the higher classification rate of the most cited circulating biomarkers, such as FibroTest, ELF, Hepascore, and FibroMeter, for the diagnosis of fibrosis stages, which were more costly compared with simple liver function tests [2,8,15]. They generally underscored the need for new NITs with better sensitivity or specificity. Several suggested that a bAUROC greater than 0.80 could be an appropriate cutoff for future qualification methods of NITs in MASLD. However, the latest international studies on recent combinations failed to demonstrate higher accuracies [15,29].

As stated in our introduction, it has been demonstrated since 2005 that it is mathematically impossible to validate an NIT with a true 80% classification rate between adjacent stages of MASLD using biopsies with a length under 25 mm [2,9,10,11,13,17]. Surprisingly, although all these reviews cited STARD, they did not realize that these tests were inappropriate (Supplementary File S8). An improvement could be to promote the utility of FibroSTARD or FibroSTARD recommendations in hepatology journals.

One review analyzed 138 studies of NITs in 46,514 cases at risk of MASLD [2]. Here, we updated this analysis, adding four comparisons published from 2023 to 2024 (Table 3) [24,30,31,32]. Due to the limited number of references, details of the 22 comparisons performed in 18 studies are provided in Table 3, and the 18 references are provided in Supplementary File S9. Sixteen studies provided the median biopsy length, but only one study used it to stratify the AUROCs. No median length > 30 mm was identified, and only one recent study used a seven-tier score. When bAUROC ≥ 0.80 was applied as a selection criterion (milestone), 12 comparisons reached this cutoff (57%): FibroTest (*n* = 4), FT-T2D (*n* = 0), ELF (*n* = 2), Hepascore (*n* = 2), FibroMeter-NAFLD (*n* = 2), and FibroMeter-v2G (*n* = 2). However, a simple adjustment by the DANA index reduced this milestone selection to only six markers of interest (27%): FibroTest (*n* = 3), FibroMeter-NAFLD (*n* = 1), FibroMeter-v2G (*n* = 1), ELF (*n* = 1), and Hepascore (*n* = 1) (Table 3).

In a road map for NITs’ assessment, several limitations were stated [8]. First, “FibroTest is less useful for early fibrosis”, an opposite conclusion than that of a more recent review [2], and by an evidence-based analysis using large biopsies [11]. The second limitation was that most data were from viral hepatitis, which was true in 2006 but not in 2024 (Table 3). ELF was considered less useful for eF, but it without evidence such as our Table 3.

In a head-to-head comparison of 335 participants including ELF, the bAUROC of 0.83 was consistent with the results of published meta-analyses regarding the diagnosis of F3 using CRN, in line with our finding that ELF-adjusted bAUROCs ranged from 0.72 to 0.80 (Table 3).

The NIMBLE study did not comment on the uncertainty of the comparator when using bAUROCs, nor did they discuss STARD criterion #15 regarding uncertainty, added in 2015, as they cited the old version [15,16]. In a recent digital pathology review, only two comments cited the biopsy length: “A 20 mm core is generally considered a best practice for assessing MASLD” and “Calculations suggested that a biopsy sample that was 22 mm in length was sufficient for a good estimation of collagen proportionate area, but stage classification is non-linear and required more tissue” [30]. More methodological examples are given in Supplementary File S5.

### 4.1. Limitations

The authors’ conflicts of interest are declared, and the patents of FibroTest or FibroTest-T2D belong to French national public organizations. We acknowledge several significant limitations that warrant external validation. This study’s design applied a post hoc analysis even though the cohorts were prospective. Proxies were highly correlated with differences in the 95% limits, but distributions should be improved. We also did not assess the uncertainty associated with the biopsy technique or different surgical methods and treatments [4,9].

We also acknowledge the cost limitations of the patented FibroTest and FibroTest-T2D when compared with simpler tests. However these tests have an advantage in the cost/benefit ratio over other NITs as they allow not only fibrosis assessment but also the assessment of MASH and steatosis grades in the same blood samples [23,31,32].

Here, we found several components with unexpected variability due to the four CFs. The significant associations observed do not prove causality, and large Mendelian randomization analyses including the four CFs are needed [2]. The results of the trajectories of liver fibrosis, inflammation, and steatosis in large cohorts at risk of MASLD according to CFs are original concepts using simultaneous proxies validated by biopsies, but external validation is also needed.

### 4.2. Strengths

Our results confirm that eF should replace bridging without cirrhosis when choosing MASLD therapy [6,7]. Using eF as a cutoff for clinically significant fibrosis permitted us to construct more sensitive NITs compared with the CRN. The 3M demonstrated an increase of FibroTest-T2D performance for the diagnosis of eF in patients with severe obesity before and after BS, which was previously observed with standard FibroTest.

We analyzed the fibrosis dynamics of NITs over a median of 9.5 years (IQR = 5.5) and 5.0 (3.4) years between biopsies. This permitted us to observe a similar FPR using FibroTest-T2D vs. histological eF changes, as observed in a trial of obeticholic acid in comparison with placebo [22].

Applying the 3M reduced the uncertainty of the NITs associated with CFs and menopause. Using proxies of eF and simultaneous steatosis and inflammatory grades permitted us to identify various trajectories according to CFs. These results will permit us to construct better prospective surveillance strategies, including forthcoming novel treatments for eF, such as resmetirom. The identification of such unusual profiles of FibroTest components already enabled the creation of warnings for eliminating COVID-19 [26] or possible Gilbert syndrome [24]. Obesity was the first CF associated with the occurrence of eF, which was significantly earlier than T2D without obesity (*p* < 0.001); eF appeared 10 years later in patients with both obesity and T2D (*p* < 0.001) [31,32]. These results warrant further focused research on topics such as the role of hormone profiles and chronic inflammation in the early increase in haptoglobin in obese women, which is also produced by adipocytes [38,39,40].

In conclusion, forthcoming studies in MASLD must add wAUROCs, stratification according to biopsy length, and use a more sensitive score than the standard CRN for credible selection. Validations of circulating biomarkers need to assess their uncertainty. FibroTest-T2D predicts fibrosis regression after bariatric surgery. Applying these methods could help avoid misinterpretations in MASLD surveillance.

## Figures and Tables

**Table 1:** A summary of the subset characteristics included for assessing the uncertainty of FibroTest-T2D according to the primary aims.

Characteristics							Three STARD Methods			FT-T2D Proxy^3^
							First Aim	Second Aim	Third Aim	Fourth Aim
Seven Subsets Reference	Disease	Number All/Biopsy/Controls	Age, Years Mean (Range or SD)	Female%	BMI, kg/m^2^ Median (Range)	T2D %	Early Fibrosis	Weighted AUROC	Liver Biopsy Sample Length	Confounding Factors of Fibrosis Progression
BARICAN Pais [6]	Bariatric surgery	55/110/0	55 (SD = 8)	62	44 (26–61)	64	Yes	Yes	Yes	Yes
QuidNash Poynard [14,23]	Type 2 diabetes	402/402/0	58 (SD = 10)	40	34 (19–54)	100	Yes	Yes	Yes	Yes
LIDO Ratziu [13]	MASLD	51/102/0	55 (31–73)	39	32 (22–45)	33	Yes	No	Yes	Yes
Fibrosis-TAGS Poynard [11]	MASLD	909/1293/4	46 (SD = 12)	39	Not available	Not available	No	Yes	Yes	Yes
UK BioBank Poynard [24]	At risk of MASLD	159,794/0/0	57 (SD = 8)	53	31 (12–75)	6	No	No	No	Yes
France- FibroTest Poynard [25]	At risk of MASLD	67,278/0/0	53 (SD = 14)	41	28 (SD = 6)	16	No	No	No	Yes
USA- FibroTest Deckmyn [26]	At risk of MASLD	72,026/0/0	56 (SD = 14)	54	31 (10–79)	22	No	No	No	Yes

**Table 2:** Performance of the new FibroTest-T2D and standard FibroTest for the diagnosis of fibrosis in QuidNash and BARICAN patients. Uncertainty is displayed according to binary AUROC (Delong method) or wAUROC (Obuchowski method), biopsy length, and choice of granularity: eF or CRN standard.

Characteristics	eF (Cutoff ≥ F1B Early Fibrosis)			CRN Standard (Cutoff ≥ F2 Bridging Fibrosis)		
**QUIDNASH COHORT N = 402**						
**Biopsy Length**	**≥Median 17 mm**	**<Median 17 mm**	**All**	**≥Median 17 mm**	**<Median 17 mm**	**All**
**Method = weighted AUROC (SD)**						
FibroTest *	0.84 (0.02)	0.80 (0.02)	0.82 (0.01)	0.87 (0.01)	0.85 (0.02)	0.86 (0.01)
*p*-value FibroTest-T2D vs. FibroTest	0.12	0.002	0.001	0.02	0.002	0.0002
*p*-value between biopsy lengths	FT-T2D < 0.001			FT-T2D < 0.001		
	FT < 0.001			FT < 0.001		
**Standard = bAUROC (95% CI)**						
N (prevalence)	*n* = 136/211 (64%)	*n* = 116/191 (61%)	*n* = 156/402 (63%)	*n* = 79/211 (37%)	*n* = 71/211 (37%)	*n* = 150/402 (37%)
FibroTest-T2D	0.72 (0.64–0.78)	0.77 (0.69–0.83)	0.74 (0.69–0.79)	0.80 (0.73–0.85)	0.74 (0.66–0.80)	0.77 (0.72–0.81)
FibroTest	0.69 (0.61–0.76)	0.67 (0.58–0.74)	0.68 (0.62–0.74)	0.74 (0.66–0.80)	0.67 (0.58–0.74)	0.70 (0.65–0.75)
BARICAN COHORT N = 110						
**Biopsy Length**	**≥Median 20 mm**	**<Median 20 mm**	**All**	**≥Median 20 mm**	**<Median 20 mm**	**All**
**Method = weighted AUROC (SD)**						
FibroTest-T2D *	0.93 (0.03) *p* < 0.001	0.88 (0.02) *p* < 0.001	0.90 (0.02) *p* < 0.001	0.91 (0.03) *p* < 0.001	0.84 (0.03) *p* < 0.001	0.87 (0.02) *p* < 0.001
FibroTest *	0.94 (0.03) *p* < 0.001	0.86 (0.03) *p* < 0.01	0.89 (0.02) *p* < 0.001	0.92 (0.03) *p* < 0.001	0.84 (0.03) *p* < 0.01	0.87 (0.02) *p* < 0.001
*p*-value FibroTest-T2D vs. FibroTest *	0.001	0.35	0.59	0.60	0.98	0.87
*p*-value between biopsy length groups	FT-T2D < 0.001			FT-T2D < 0.001		
	FT < 0.001			FT < 0.001		
Standard = bAUROC (95% CI),						
N (prevalence)	*n* = 8/73 (11%)	*n* = 12/37 (17%)	*n* = 18/110 (27%)	*n* = 11/43 (26%)	*n* = 16/67 (24%)	*n* = 27/110 (28%)
FibroTest-T2D	0.84 (0.64–0.93)	0.72 (0.47–0.90)	0.78 (0.63–0.87)	0.84 (0.65–0.93)	0.83 (0.67–0.92)	0.83 (0.72–0.90)
FibroTest	0.80 (0.46–0.94)	0.49 (0.25–0.68)	0.67 (0.50–0.80)	0.78 (0.57–0.89)	0.76 (0.57–0.88	0.77(0.63–0.86)

* *p*-value for the bAUROC and wAUROC methods. eF is the earliest stage of the four clinically significant fibrosis stages: F1B, F2, F3, and F4. F0 includes no fibrosis, F1A, and F1C [8,25]. wAUROC: weighted area under the receiver operating characteristic curve (Obuchowski measure). FT-T2D proxy: Serum proxy of eF stages constructed and validated in large population subsets.

**Table 3:** Uncertainty of the four most cited circulating fibrosis markers for the diagnosis of fibrosis stages F3/F4 vs. F0/F1/F2 in MASLD. Sequential combinations were excluded. Using non-adjusted binary AUROCs, 12 studies achieved the 0.80 cutoff with a high risk of spectrum bias, but this number was reduced to only 6 when adjusted for spectrum effect (In bold).

22 Comparisons in 18 Published Studies Author Year	N	eF	CRN F0/F1/F2/F3/F4	DANA Index	Adjusted AUROC F3F4 vs. F0F1F2	Weighted AUROC	Binary AUROC Standard F3/F4 vs. F0/F1/F2	Biopsy Length Median (mm)
Uniform spectrum model	100		20/20/20/20/20	2.50	0.800	0.800	**>0.800**	>30
**FibroTest**								
Ratziu 2006 [33] first	97	0	26/40/15/12/4	2.39	**0.910**	0.878	**0.810**	18
Ratziu 2006 [33] validation	170	0	76/54/31/9/0	2.28	**0.873**	0.920	**0.920**	20
Lassailly 2011 [34]	288	0	170/98/13/2/5	3.27	**0.911**	0.847	**0.971**	NA
Adams 2011 *	242	0	87/58/44/30/23	2.38	0.784	NA	**0.802**	16
Munteanu 2016 [35]	600	0	122/184/140/121/33	2.17	0.744	0.878	0.749	20
Boursier 2016 [17] *^,^,w^	452	0	41/117/120/114/58	2.06	0.735	0.722	0.734	27
Bril 2020 *	151	0	38/63/25/19/6	2.34	0.722	NA	0.720	NA
Poynard 2023 [23] ^2,^,l,r,w^	402	1	117/66/63/85/71	2.67	0.789	0.842	0.709	17
**FT-T2D**								
Poynard 2023 [23] ^2,^,b,l,r,w^	402	1	117/66/63/85/71	2.67	0.789	0.879	0.774	17
**ELF**								
Miele 2017 *	82	0	6/32/29/5/10	2.32	0.759	NA	**0.948**	>16
Anstee 2019 *	3202	0	246/276/418/979/128	2.38	0.764	NA	0.800	22
Guillaume 2019 ^^,w,^*	417	0	38/98/114/135/32	1.89	0.720	0.764	0.793	29
Arai 2024 [36] ^r^	1228	0	214/411/327/237/39	2.02	**0.803**	NA	**0.828**	NA
**Hepascore**								
Adams 2011 *^,^^	242	0	87/58/44/30/23	2.38	0.788	NA	**0.814**	16
Bertot 2023 [37] ^r^	271	0	101/67/20/36/47	3.00	**0.842**	NA	**0.880**	NA
Boursier 2016 [17] ^2,^,w^	452	0	41/117/120/114/58	2.06	0.735	0.765	0.778	
**FibroMeter NAFLD**								
Cales 2009 *	235	0	102/68/21/19/25	2.99	**0.889**	NA	**0.928**	30
Aykut 2014 ^t,^*	88	0	23/21/17/27/9	2.35	0.761	NA	**0.937**	NA
Boursier 2016 [17] ^^,w,^	452	0	41/117/120/114/58	2.06	0.735	0.886	0.759	27
Subasi 2015 ^t,^*	142	0	40/50/22/20/10	2.49	0.774	NA	0.761	20
**FibroMeter V2G**								
Boursier 2016 [17] ^2,^,w^	452	0	41/117/120/114/58	2.06	0.735	0.798	**0.817**	27
Guillaume 2019 ^^,w,^*	417	0	38/98/114/135/32	1.89	0.720	0.763	**0.804**	29

DANA = Difference between Advanced and Non-Advanced fibrosis. NA: Not available. ^^^ Head-to-head comparison (*n* = 8). ^2^ Analysis in intention-to-diagnose (*n* = 2). ^b^ eF early fibrosis (*n* = 2) is the earliest stage of the four clinically significant fibrosis stages: F1B, F2, F3, and F4. F0 includes no fibrosis, F1A, and F1C [7,20]. ^l^ Binary AUROC (Delong test) stratified according to biopsy length (*n* = 2). ^r^ Recently published after 2021 (*n* = 4). ^w^ wAUROC (*n* = 8) weighted area under the receiver operating characteristic curve (Obuchowski measure). ^t^ Two studies sharing the same patients, one excluding VCTE (Aykut 2014). * Nine references not cited in the article are listed in Supplementary File S9 (*n* = 9).

## Data Availability

The original contributions presented in this study are included in the article and Supplementary Material. Further inquiries can be directed to the corresponding author.

## Supplementary Materials

The following supporting information can be downloaded at: https://www.mdpi.com/article/10.3390/diagnostics15101253/s1, Table S1: Comparison between included and non-included BARICAN participants at the date of surgery. Table S2: Comparison of major confounding factors in the US FibroTest, France FibroTest, and the UK Biobank subsets. Table S3: Component details of FibroTest-T2D (FT-T2D) a combination for the diagnosis of fibrosis stages (FibroTest), grades (MashTest), and steatosis grades (SteatoTest) in QuidNash participants. Table S4. Characteristics of UK biobank participants, per sex. Table S5. Characteristics of UK Biobank participants at risk of MASLD with or without bariatric surgery history. Table S6. Factors associated with menopause in UK Biobank women participants. Table S7. Reference values of fibrosis area assessed by CRN-5-tier score and image analysis of Fibrosis-TAGS. Table S8. Multivariate regression analysis of factors associated with menopause in UK Biobank women participants. Figure S1. BARICAN participants (included and not included) flow chart. Figure S2. UK Biobank flow chart of patients not included in the general population. Figure S3. FibroTest-T2D progression/regression rates (FPR) in BARICAN participants before BS (biopsy-1), between BS and follow-up (biopsy-2), and after biopsy-2 according to age and sex. A total of 355 FibroTest-T2D were assessed, a median of four per patient. Figure S4. Apolipoprotein A1 in France FibroTest, US FibroTest, and UK Biobank subsets according to sex, T2D, and BMI. Figure S5. Haptoglobin in France FibroTest and US FibroTest subset according to sex, glucose level, and BMI. Figure S6. Alpha-2 Macroglobulin in France FibroTest vs. US FibroTest subset according to sex, T2D, and BMI. Figure S7. Men’s fibrosis progression rates in the US and France populations at risk of MASLD. File S1. Definitions of accuracy and uncertainty in the literature and in STARD or Liver-FibroSTARD statements in fibrosis biomarker studies. File S2. Ethics. File S3. BARICAN subset design for validating bariatrics surgery. File S4. Summary of QuidNash protocol for validating FT-T2D. File S5. Method details and examples. File S6. Bland–Altman plots. File S7. Sensitivity analyses. File S8. Updated meta-analysis overview. File S9. References of the 9 studies from Table 3.
