# Supplementary material for: Three Neglected STARD Criteria Reduce the Uncertainty of the Liver Fibrosis Biomarker FibroTest-T2D in Metabolic Dysfunction-Associated Steatotic Liver Disease (MASLD)
Source: Diagnostics (Basel). 2025 May 15;15(10):1253. doi: 10.3390/diagnostics15101253 (PMC12110081; doi:10.3390/diagnostics15101253)
Supplement: Supplementary file 1 [file diagnostics-15-01253-s001.zip › Supp Files FT-T2D Diagnostics TP.pdf]

Three neglected STARD criteria reduce the uncertainty of the liver fibrosis biomarker FibroTest-T2D in metabolic dysfunction-associated steatotic liver disease (MASLD)

SUPPLEMENTARY MATERIAL: TABLES, FIGURES AND FILES

SUPPLEMENTARY TABLES

Supplementary-Table-S1. Comparison between included and non-included at the date of surgery in Barican participants
Supplementary-Table-S2. Comparison of major confounding factors in the USA-FibroTest, the France-FibroTest and the UK-Biobank subsets.
Supplementary-Table-S3. Components details of FibroTest-T2D (FT-T2D) a combo for the diagnosis of fibrosis stages (FibroTest), grades (MashTest) and steatosis grades (SteatoTest), in QuidNash participants.
Supplementary-Table-S4. Characteristics of UK biobank participants, per sex
Supplementary-Table-S5. Characteristics of UK Biobank participants at risk of MASLD with or without bariatric surgery history
Supplementary-Table-S6. Factors associated with menopause in UK Biobank women participants
Supplementary-Table-S7. Reference values of fibrosis area assessed by CRN-5-tier score and image analysis in Fibrosis-TAGS
Supplementary-Table-S8. Multivariate regression analysis of factors associated with menopause in UK Biobank women participants

SUPPLEMENTARY FIGURES

Supplementary-Figure-S1. BARICAN participants flow-chart, included and not included.
Supplementary-Figure-S2. UK-Biobank flow-chart of patients not included in the general population.
Supplementary-Figure-S3. FibroTest-T2D progression/regression rates (FPR) in BARICAN participants before BS (biopsy-1), between BS and follow-up (biopsy-2), and after biopsy-2 according to age and sex A total of 355 FibroTest-T2D were assessed, a median of four per patient.
Supplementary-Figure-S4. Apolipoprotein A1 in France-FibroTest, USA-FibroTest and UK-Biobank subsets according to sex, T2D and BMI.
Supplementary-Figure-S5. Haptoglobin in France-FibroTest and USA-FibroTest subset according to sex, glucose and BMI.
Supplementary-Figure-S6. Alpha-2 Macroglobulin in France-FibroTest vs USA-FibroTest subset according to sex, T2D and BMI
Supplementary-Figure-S7. Men Fibrosis progression rates in USA and France populations at risk of MASLD

SUPPLEMENTARY FILES

Supplementary-File-S1. Definitions of accuracy and uncertainty in literature and in STARD or Liver-FibroSTARD statements in fibrosis biomarkers studies
Supplementary-File-S2. Ethics
Supplementary-File-S3. BARICAN subset design for validating bariatrics surgery
Supplementary-File-S4. Summary of QuidNash protocol for validating FT-T2D
Supplementary-File-S5. Methods details and examples
Supplementary-File-S6. Bland-Altman plots
Supplementary-File-S7. Sensitivity analyses.
Supplementary-File-S8. Updated meta-analysis overview
Supplementary-File-S9. References of the 9 studies of Table 3

Supplementary-Table-S1. Comparison between included and non-included at the date of surgery in the Barican cohort

**Characteristics**	**Included n=55**	**Non-included n=11**	**P-value**
Age years (mean ± SD)	51 ± 8	55 ± 13	0.18
Sex (females, %)	61	64	0.88
Type of surgery (RYGB, %)	78	91	0.61
Weight (kg, mean ± SD)	128 ± 22	99 ± 10	0.0001
BMI (kg/m^2, mean ± SD)	45 ± 6	35 ± 9	0.004
Obesity class <30/30+/35+/40+/45+ (%)	0/2/16/35/47	36/9/9/18/27	0.001
Type 2 Diabetes (%)	80	70	0.60
Arterial hypertension (%)	81	73	0.53
Obstructive sleep apnea (%)	80	73	0.29
**Histological score EPOS F3-F6 (%)**	**29**	**20**	**0.60**
**Histological SAF scores SAF**			
Fibrosis stage F0/F1/F2/F3/F4 (%)	33/31/13/14/9	50/20/10/10/10	0.54
grade A0/A1/A2/A3/A4 (%)	35/25/13/22/5	73/9/9/9/0	0.78
Steatosis grade S0/S1/S2/S3 (%)	0/7/44/49	0/22/44/33	0.14
Biopsy length (mm, median, range)	20 (5-100)	25 (10-38)	0.79
**FibroTest-T2d indexes**			
FibroTest-T2d mean ± SD)	0.46 ± 0.25	Not applicable	Not applicable
MashTest-T2d mean ± SD)	0.44 ± 0.23	Not applicable	Not applicable
SteatoTest-T2d mean ± SD)	0.96 ± 0.06	Not applicable	Not applicable

Abbreviations: RYGB, Roux-en-Y gastric bypass. EPoS, Elucitading Pathways of Steatohepatitis.

Supplementary-Table-S2. Comparison of major confounding factors in the USA-FibroTest and the France-FibroTest subsets.

**Characteristics**	**UK-BioBank**	**USA-FibroTest**	**France-FibroTest**
Number	159,794	72,026	67,278
Unit	median (IQR)	mean ± SD	mean ± SD
Age years	59 (52-64)	51 ± 8	55 ± 13
Weight kg	85 (74-96)	128 ± 22	99 ± 10
BMI (kg/m^2)	30 (27-33)	45 ± 6	35 ± 9
Sex females (%)	76,695 (48)	38,842 (53.9)	27,743 (41.2)
Clinical type 2 diabetes (T2D %)	15,667 (9.8)	NA	NA
T2D proxy, glucose >7mmol/L	11,522 (7.2)	16,147 (22.4)	10,791 (16.1)
**Fibrosis stages 7-tear n (%)**			
F0	NA	107 (0.2)	81 (0.1)
F1	NA	11,962 (16.8)	9,261 (13.8)
F2	NA	18,607 (25.8)	17,108 (25.4)
F3	NA	10,467 (17.9)	12,451 (18.5)
F4	NA	12,918 (14.5)	9,638 (14,3)
F5	NA	8,660 (12.0)	8,654 (12.9)
F6	NA	9,305(12.9)	10,068 (15.0)
BMI classes			
<27	47,748 (29)	18,013 (25.0)	36,225 (53.8)
27-29	33,693 (21)	13,055 (18.1)	12,622 (18.8)
30-34	58,251 (36)	19,586 (27,2)	11,316 (16.8)
35-39	16,210 (10)	11,847 (16,5)	4,047 (6.0)
40-44	4,397 (2,8)	5,501 (7,6)	1,747 (2,6)
>= 45	1,495 (0.9)	4,024 (5,6)	1,321 (2,0)

Supplementary-Table-S3. Components details of FibroTest-T2D (FT-T2D) a combo for the diagnosis of fibrosis stages (FibroTest), grades (MashTest) and steatosis grades (SteatoTest), in QuidNash participants.

	**Optimized FibroTest components for T2D (FT-T2D)**		**Regular FibroMax, Nash-FibroSure**	
	**Female**	**Male**	**Female**	**Male**
**Fibrosis**				
Test name	FibroTest-T2D	FibroTest-T2D	FibroTest	FibroTest
Components	n=5	n=5	n=6	n=6
Age	yes	yes	yes	yes
A2M	yes	yes	yes	yes
ApoA1	yes	yes	yes	yes
Haptoglobin	yes	yes	yes	yes
GGT	yes	yes	yes	yes
Bilirubin	no	no	yes	yes
**MASH**				
Test name	NashTest-T2D	NashTest-T2D	NashTest	NashTest
Components	n=5	n=6	n=9	n=9
Age	yes	yes	yes	yes
A2M	yes	yes	yes	yes
ApoA1	no	no	yes	yes
Haptoglobin	no	no	yes	yes
GGT	no	yes	yes	yes
Total bilirubin	yes	yes	yes	yes
ALT	no	no	yes	yes
AST	yes	yes	yes	yes
TGL	no	no	yes	yes
HbA1c	**yes**	**yes**	no	no
**Steatosis**				
Test name	SteatoTest-T2D	SteatoTest-T2D	SteatoTest	SteatoTest
Components	n=9	n=7	n=11	n=11
Age	yes	yes	yes	yes
A2M	no	no	yes	yes
ApoA1	yes	yes	yes	yes
Haptoglobin	yes	**no**	yes	yes
GGT	**no**	yes	yes	yes
Total bilirubin	yes	yes	yes	yes
ALT	yes	yes	yes	yes
AST	no	no	yes	yes
TGL	yes	yes	yes	yes
BMI	**yes**	no	no	no
HbA1c	**yes**	no	no	no
Glucose	no	no	yes	yes
Total cholesterol	yes	yes	yes	yes

Supplementary-Table-S4. Characteristics of UK biobank participants, per sex.

	Females			Males		
**Characteristic**	**At risk of MASLD**, N = 76,695	**History of barriatric surgery**, N = 437	**p-value**[1]	**At risk of MASLD**, N = 83,099	**History of barriatric surgery**, N = 244	**p-value**[1]
**Age (year), Median (IQR)**	59 (53 – 64)	53 (47 – 58)	**<0.001**	59 (52 – 64)	57 (51 – 62)	**0.004**
**Weight (kg), Median (IQR)**	78 (68 – 88)	106 (86 – 122)	**<0.001**	91 (81 – 101)	110 (86 – 134)	**<0.001**
**Height (m), Median (IQR)**	162 (158 – 166)	161 (158 – 166)	0.82	175 (171 – 180)	175 (170 – 179)	0.33
**Alcohol intake, n (%)**			**<0.001**			**<0.001**
low	31,781 (41)	255 (58)		17,820 (21)	99 (41)	
mild or high	44,914 (59)	182 (42)		65,279 (79)	145 (59)	
**Average alcohol intake (g), Median (IQR)**	0.43 (0.00 – 1.14)	0.03 (0.00 – 0.86)	**<0.001**	1.29 (0.43 – 2.57)	0.71 (0.00 – 2.18)	**<0.001**
**Smoker status, n (%)**			**0.001**			0.99
never	44,132 (58)	214 (49)		36,608 (44)	107 (44)	
current	6,457 (8.5)	45 (10)		9,918 (12)	30 (12)	
previous	25,796 (34)	176 (40)		36,232 (44)	106 (44)	
(Missing)	310	2		341	1	
**Body mass index (kg/m2), Median (IQR)**	30.2 (26.1 – 33.4)	40.6 (33.4 – 47.1)	**<0.001**	29.7 (26.8 – 32.2)	35.8 (28.8 – 43.2)	**<0.001**
**Body mass index (kg/m2), n (%)**			**<0.001**			
<27	23,452 (31)	56 (13)		22,296 (27)	51 (21)	
27-<30	12,902 (17)	26 (5.9)		20,791 (25)	34 (14)	
30-<35	27,145 (35)	43 (9.8)		31,106 (37)	28 (11)	
35-<40	9,315 (12)	77 (18)		6,895 (8.3)	40 (16)	
40-<45	2,877 (3.8)	91 (21)		1,520 (1.8)	44 (18)	
45+	1,004 (1.3)	144 (33)		491 (0.6)	47 (19)	
**C-Reactive protein (mg/l), Median (IQR)**	2.2 (1.1 – 4.4)	4.1 (2.0 – 9.7)	**<0.001**	1.63 (0.87 – 3.08)	2.66 (1.08 – 5.50)	**<0.001**
(Missing)	138	1		154	0	
**ALT (UI/l), Median (IQR)**	21 (16 – 29)	21 (16 – 28)	0.39	28 (21 – 38)	25 (19 – 34)	**<0.001**
**AST (UI/l), Median (IQR)**	24 (21 – 29)	23 (20 – 28)	**<0.001**	28 (24 – 33)	27 (22 – 32)	**0.015**
**Glucose (mmol/Ll, Median (IQR)**	5.00 (4.67 – 5.44)	5.10 (4.68 – 5.67)	**0.008**	5.04 (4.67 – 5.53)	5.20 (4.79 – 5.97)	**<0.001**
(Missing)	62	0		85	0	

[1] Wilcoxon rank sum test; Pearson's Chi-squared test; Fisher's exact test

Supplementary-Table-S4: Characteristics of UK biobank participants per sex, **continued**

	Females			Males		
**Characteristic**	**At risk of MASLD**, N = 76,695	**History of barriatric surgery**, N = 437	**p-value**[1]	**At risk of MASLD**, N = 83,099	**History of barriatric surgery**, N = 244	**p-value**[1]
**Glycated Haemoglobin Hb1Ac (%), Median (IQR)**	5.45 (5.22 – 5.70)	5.49 (5.24 – 5.93)	**<0.001**	5.44 (5.20 – 5.75)	5.64 (5.26 – 6.13)	**<0.001**
**Platelet Count (10^9 cells/l), Median (IQR)**	267 (231 – 307)	280 (235 – 320)	**<0.001**	234 (202 – 270)	234 (196 – 271)	0.73
**Fib-4 index, Median (IQR)**	1.16 (0.92 – 1.47)	0.98 (0.76 – 1.28)	**<0.001**	1.30 (1.02 – 1.66)	1.27 (1.02 – 1.62)	0.86
**Creatinine (µmol/l), Median (IQR)**	64 (57 – 71)	62 (55 – 70)	**0.003**	80 (73 – 89)	77 (68 – 86)	**<0.001**
(Missing)	4	0		10	0	
**Total Cholesterol (mmol/l), Median (IQR)**	5.84 (5.06 – 6.62)	5.39 (4.63 – 6.16)	**<0.001**	5.35 (4.54 – 6.18)	4.85 (4.17 – 5.60)	**<0.001**
**HDL Cholesterol (mmol/l), Median (IQR)**	1.45 (1.24 – 1.71)	1.30 (1.13 – 1.52)	**<0.001**	1.19 (1.02 – 1.39)	1.13 (0.97 – 1.34)	**0.005**
**Apolipoprotein-A1 (g/l), Median (IQR)**	1.57 (1.41 – 1.74)	1.47 (1.33 – 1.64)	**<0.001**	1.38 (1.25 – 1.54)	1.36 (1.23 – 1.50)	**0.015**
**Albumin (g/l), Median (IQR)**	44.74 (43.03 – 46.47)	43.28 (41.75 – 44.78)	**<0.001**	45.54 (43.81 – 47.27)	44.22 (42.24 – 46.14)	**<0.001**
(Missing)	24	0		28	0	
**Total Bilirubin (µmol/l), Median (IQR)**	7.10 (5.78 – 8.94)	6.67 (5.25 – 8.37)	**<0.001**	9.1 (7.3 – 11.6)	8.2 (6.5 – 10.0)	**<0.001**
(Missing)	8	0		17	0	
**Direct Bilirubin (µmol/l), Median (IQR)**	1.44 (1.21 – 1.81)	1.49 (1.22 – 1.85)	0.20	1.81 (1.46 – 2.33)	1.68 (1.36 – 2.20)	**0.008**
(Missing)	17,216	120		5,156	20	
**GGT (UI/l), Median (IQR)**	27 (19 – 45)	29 (20 – 45)	0.33	42 (29 – 69)	39 (27 – 58)	**0.004**
**Triglycerides (mmol/l), Median (IQR)**	1.59 (1.15 – 2.21)	1.66 (1.24 – 2.29)	0.073	1.90 (1.33 – 2.72)	1.76 (1.21 – 2.52)	**0.033**
**Oestradiol (pmol/l), Median (IQR)**	369 (248 – 590)	327 (245 – 499)	0.15	204 (189 – 231)	218 (193 – 256)	0.18
(Missing)	63,192	292		75,708	207	
**Testosterone (nmol/l), Median (IQR)**	1.04 (0.74 – 1.41)	1.11 (0.82 – 1.55)	**0.002**	10.9 (8.9 – 13.3)	9.9 (7.8 – 12.7)	**<0.001**
(Missing)	12,268	64		762	2	
**Had menopause, n (%)**	49,869 (80)	191 (60)	**<0.001**	0 (NA)	0 (NA)	
(Missing)	14,054	120		83,099	244	
**Age of menopause (year), Median (IQR)**	50 (47 – 53)	50 (45 – 52)	**<0.001**	NA (NA – NA)	NA (NA – NA)	
(Missing)	30,201	257		83,099	244	

[1] Wilcoxon rank sum test; Pearson's Chi-squared test; Fisher's exact test

Supplementary-Table-S4: Characteristics of UK biobank participants per sex, **continued**

	Females			Males		
**Characteristic**	**At risk of MASLD**, N = 76,695	**History of barriatric surgery**, N = 437	**p-value**[1]	**At risk of MASLD**, N = 83,099	**History of barriatric surgery**, N = 244	**p-value**[1]
**Age started hormone-replacement therapy (HRT), Median (IQR)**	48 (44 – 50)	44 (39 – 49)	**<0.001**	NA (NA – NA)	NA (NA – NA)	
(Missing)	46,886	299		83,099	244	
**Age last used hormone-replacement therapy (HRT), Median (IQR)**	55 (50 – 58)	50 (45 – 55)	**<0.001**	NA (NA – NA)	NA (NA – NA)	
(Missing)	51,170	326		83,099	244	
**Had hysterectomy, n (%)**	6,449 (9.8)	34 (9.7)	0.94	0 (NA)	0 (NA)	
(Missing)	10,945	86		83,099	244	
**Age at hysterectomy (year), Median (IQR)**	43 (38 – 49)	40 (35 – 45)	**<0.001**	NA (NA – NA)	NA (NA – NA)	
(Missing)	59,794	318		83,099	244	
**Overall Death, n (%)**	4,509 (5.9)	59 (14)	**<0.001**	8,591 (10)	70 (29)	**<0.001**
**Liver-related Death, n (%)**	94 (0.1)	3 (0.7)	**0.018**	228 (0.3)	7 (2.9)	**<0.001**
**Liver Cancer Death, n (%)**	23 (<0.1)	1 (0.2)	0.13	46 (<0.1)	0 (0)	>0.99

[1] Wilcoxon rank sum test; Pearson's Chi-squared test; Fisher's exact test

[1] Odds Ratio (95% confidence interval) [2] q-value is significance after adjustment on the number of comparisons.

Supplementary-Table-S5. Characteristics of UK Biobank participants at risk of MASLD with or without bariatric surgery history.

**Characteristic**	**At risk of MASLD**, N = 159,794	**History of barriatric surgery**, N = 681	**p-value**[1]
Age (year), Median (IQR)	59 (52 – 64)	54 (48 – 60)	**<0.001**
Weight (kg), Median (IQR)	85 (74 – 96)	107 (86 – 126)	**<0.001**
Height (m), Median (IQR)	169 (162 – 176)	166 (160 – 173)	**<0.001**
Alcohol intake, n (%)			**<0.001**
low	49,601 (31)	354 (52)	
mild or high	110,193 (69)	327 (48)	
Average alcohol intake (g), Median (IQR)	0.86 (0.00 – 1.86)	0.20 (0.00 – 1.14)	**<0.001**
Smoker status, n (%)			0.21
never	80,740 (51)	321 (47)	
current	16,375 (10)	75 (11)	
previous	62,028 (39)	282 (42)	
(Missing)	651	3	
Body mass index (kg/m2), Median (IQR)	30.0 (26.5 – 32.7)	39.3 (30.2 – 45.9)	**<0.001**
Body mass index (kg/m2), n (%)			**<0.001**
<27	45,748 (29)	107 (16)	
27-<30	33,693 (21)	60 (8.8)	
30-<35	58,251 (36)	71 (10)	
35-<40	16,210 (10)	117 (17)	
40-<45	4,397 (2.8)	135 (20)	
45+	1,495 (0.9)	191 (28)	
C-Reactive protein (mg/l), Median (IQR)	1.9 (1.0 – 3.7)	3.4 (1.5 – 7.7)	**<0.001**
(Missing)	292	1	
ALT (UI/l), Median (IQR)	24 (18 – 34)	22 (17 – 30)	**<0.001**
AST (UI/l), Median (IQR)	26 (22 – 31)	25 (20 – 30)	**<0.001**
Glucose (mmol/LI, Median (IQR)	5.02 (4.67 – 5.49)	5.12 (4.72 – 5.80)	**<0.001**
(Missing)	147	0	
Glycated Haemoglobin Hb1Ac (%), Median (IQR)	5.45 (5.21 – 5.73)	5.53 (5.25 – 6.01)	**<0.001**
Platelet Count (10^9 cells/l), Median (IQR)	249 (214 – 289)	261 (221 – 308)	**<0.001**
Fib-4 index, Median (IQR)	1.23 (0.96 – 1.57)	1.09 (0.84 – 1.40)	**<0.001**
Creatinine (µmol/l), Median (IQR)	72 (63 – 83)	67 (58 – 78)	**<0.001**
(Missing)	14	0	
Total Cholesterol (mmol/l), Median (IQR)	5.59 (4.78 – 6.41)	5.17 (4.39 – 6.00)	**<0.001**

[1] Wilcoxon rank sum test; Pearson's Chi-squared test; Fisher's exact test

In comparison with participants at risk of MASLD, those with BS were significantly (all P< .001) younger, had lower alcohol consumption, higher serum HbA1c, glucose, C-reactive protein, and platelet count but lower ALT and AST transaminases, FIB4, creatinine, apoA1, and total cholesterol.

Supplementary-Table-S5. Characteristics of UK Biobank participants at risk of MASLD with or without bariatric surgery history **continued.**

**Characteristic**	**At risk of MASLD**, N = 159,794	**History of barriatric surgery**, N = 681	**p-value**[1]
HDL Cholesterol (mmol/l), Median (IQR)	1.31 (1.11 – 1.56)	1.24 (1.05 – 1.46)	**<0.001**
Apolipoprotein-A1 (g/l), Median (IQR)	1.47 (1.31 – 1.65)	1.42 (1.27 – 1.59)	**<0.001**
Albumin (g/l), Median (IQR)	45.16 (43.41 – 46.91)	43.56 (41.95 – 45.32)	**<0.001**
(Missing)	52	0	
Total Bilirubin (µmol/l), Median (IQR)	8.1 (6.4 – 10.4)	7.1 (5.7 – 9.1)	**<0.001**
(Missing)	25	0	
Direct Bilirubin (µmol/l), Median (IQR)	1.64 (1.31 – 2.12)	1.58 (1.28 – 2.00)	**0.004**
(Missing)	22,372	140	
GGT (UI/l), Median (IQR)	35 (23 – 59)	32 (22 – 50)	**<0.001**
Triglycerides (mmol/l), Median (IQR)	1.74 (1.23 – 2.47)	1.69 (1.23 – 2.35)	0.16
Oestradiol (pmol/l), Median (IQR)	264 (204 – 456)	287 (222 – 452)	**0.039**
(Missing)	138,900	499	
Testosterone (nmol/l), Median (IQR)	7.4 (1.1 – 11.4)	1.7 (1.0 – 8.9)	**<0.001**
(Missing)	13,030	66	
Had menopause, n (%)	49,869 (80)	191 (60)	**<0.001**
(Missing)	97,153	364	
Age of menopause (year), Median (IQR)	50 (47 – 53)	50 (45 – 52)	**<0.001**
(Missing)	113,300	501	
Age started hormone-replacement therapy (HRT), Median (IQR)	48 (44 – 50)	44 (39 – 49)	**<0.001**
(Missing)	129,985	543	
Age last used hormone-replacement therapy (HRT), Median (IQR)	55 (50 – 58)	50 (45 – 55)	**<0.001**
(Missing)	134,269	570	
Had hysterectomy, n (%)	6,449 (9.8)	34 (9.7)	0.94
(Missing)	94,044	330	
Age at hysterectomy (year), Median (IQR)	43 (38 – 49)	40 (35 – 45)	**<0.001**
(Missing)	142,893	562	
Overall Death, n (%)	13,100 (8.2)	129 (19)	**<0.001**
Liver-related Death, n (%)	322 (0.2)	10 (1.5)	**<0.001**
Liver Cancer Death, n (%)	69 (<0.1)	1 (0.1)	0.26

[1] Wilcoxon rank sum test; Pearson’s Chi-squared test; Fisher’s exact test

Supplementary-Table-S6. Factors associated with menopause in UK Biobank women participants

Characteristic	**Menopause**, N = 50,060	**No menopause**, N = 12,898	**p-value**[1]
Age (year), Median (IQR)	61 (57 – 65)	46 (43 – 50)	**<0.001**
Weight (kg), Median (IQR)	76 (67 – 87)	83 (72 – 94)	**<0.001**
Height (m), Median (IQR)	161 (157 – 165)	163 (159 – 167)	**<0.001**
Alcohol intake, n (%)			0.69
low	20,380 (41)	5,226 (41)	
mild or high	29,680 (59)	7,672 (59)	
Average alcohol intake (g), Median (IQR)	0.43 (0.00 – 1.14)	0.57 (0.00 – 1.29)	**<0.001**
Smoker status, n (%)			**<0.001**
never	28,076 (56)	8,097 (63)	
current	3,829 (7.7)	1,351 (10)	
previous	17,933 (36)	3,422 (27)	
(Missing)	222	28	
Body mass index (kg/m2), Median (IQR)	29.7 (25.8 – 32.9)	31.2 (27.2 – 34.7)	**<0.001**
Body mass index (kg/m2), n (%)			**<0.001**
<27	16,652 (33)	3,120 (24)	
27-<30	9,054 (18)	1,449 (11)	
30-<35	16,692 (33)	5,331 (41)	
35-<40	5,406 (11)	2,017 (16)	
40-<45	1,647 (3.3)	664 (5.1)	
45+	609 (1.2)	317 (2.5)	
C-Reactive protein (mg/l), Median (IQR)	2.1 (1.1 – 4.2)	2.2 (1.0 – 4.5)	0.47
(Missing)	100	15	
ALT (UI/l), Median (IQR)	21 (16 – 29)	19 (14 – 26)	**<0.001**
AST (UI/l), Median (IQR)	25 (21 – 30)	22 (19 – 26)	**<0.001**
Glucose (mmol/Ll, Median (IQR)	5.04 (4.71 – 5.49)	4.87 (4.55 – 5.26)	**<0.001**
(Missing)	43	8	
Glycated Haemoglobin Hb1Ac (%), Median (IQR)	5.49 (5.27 – 5.74)	5.25 (5.03 – 5.49)	**<0.001**
Platelet Count (10^9 cells/l), Median (IQR)	263 (228 – 302)	280 (242 – 323)	**<0.001**
Fib-4 index, Median (IQR)	1.25 (1.02 – 1.55)	0.84 (0.69 – 1.04)	**<0.001**
Creatinine (µmol/l), Median (IQR)	64 (58 – 71)	63 (57 – 69)	**<0.001**
(Missing)	4	0	
Total Cholesterol (mmol/l), Median (IQR)	5.93 (5.13 – 6.72)	5.49 (4.84 – 6.19)	**<0.001**
HDL Cholesterol (mmol/l), Median (IQR)	1.48 (1.27 – 1.74)	1.37 (1.17 – 1.61)	**<0.001**
Apolipoprotein-A1 (g/l), Median (IQR)	1.59 (1.43 – 1.76)	1.49 (1.34 – 1.66)	**<0.001**
Albumin (g/l), Median (IQR)	44.83 (43.14 – 46.55)	44.32 (42.58 – 46.03)	**<0.001**
(Missing)	18	5	
Total Bilirubin (µmol/l), Median (IQR)	7.2 (5.9 – 9.0)	6.9 (5.5 – 8.9)	**<0.001**
(Missing)	6	1	
Direct Bilirubin (µmol/l), Median (IQR)	1.44 (1.21 – 1.80)	1.47 (1.21 – 1.86)	**<0.001**
(Missing)	10,761	3,123	
GGT (UI/l), Median (IQR)	28 (20 – 46)	24 (17 – 38)	**<0.001**
Triglycerides (mmol/l), Median (IQR)	1.61 (1.17 – 2.22)	1.40 (1.01 – 1.97)	**<0.001**
Oestradiol (pmol/l), Median (IQR)	262 (206 – 409)	411 (278 – 646)	**<0.001**
(Missing)	47,470	4,670	
Testosterone (nmol/l), Median (IQR)	1.01 (0.72 – 1.38)	1.20 (0.86 – 1.59)	**<0.001**
(Missing)	8,436	1,007	
Had menopause, n (%)	50,060 (100)	0 (0)	**<0.001**
Age of menopause (year), Median (IQR)	50 (47 – 53)	NA (NA – NA)	
(Missing)	3,386	12,898	
Age started hormone-replacement therapy (HRT), Median (IQR)	49 (45 – 51)	47 (43 – 50)	**<0.001**
(Missing)	28,361	12,328	
Age last used hormone-replacement therapy (HRT), Median (IQR)	55 (51 – 58)	48 (45 – 52)	**<0.001**
(Missing)	30,553	12,540	
Had hysterectomy, n (%)	6,364 (13)	69 (0.5)	**<0.001**
(Missing)	49	11	
Age at hysterectomy (year), Median (IQR)	45 (40 – 52)	39 (35 – 45)	**<0.001**
(Missing)	43,871	12,832	
Overall Death, n (%)	3,459 (6.9)	254 (2.0)	**<0.001**
Liver-related Death, n (%)	61 (0.1)	12 (<0.1)	0.39
Liver Cancer Death, n (%)	18 (<0.1)	0 (0)	**0.035**

[1] Wilcoxon rank sum test; Pearson's Chi-squared test; Fisher's exact test

Supplementary-Table-S7. Reference values of fibrosis area assessed by 5-tier CRN score and VCTE analysis in 8 Fibrosis-TAGS participants with large biopsies

From the 25 Fibrosis-TAGS participants with large liver biopsies, the posthoc analysis included 8 subjects with contemporary Fibrotest and VCTE and normal liver histology (n=4), or MASLD (n=4). The characteristics of these included participants were bold in green.

Case no.	Age (y)	Gender	Diagnosis	METAVIR score	FibroTest	FibroScan (kPa)	% AF[a] mean (n)
No fibrosis[b]							
1	63	Male	Colon cancer	F0	0.22	9.3	1.6 (246)
2	68	Male	Colon cancer	F0	0.25	5.3	3.9 (192)
3	40	Male	Adenoma	F0	0.26	4.4	4.1 (150)
4	44	Female	Colon cancer	F0	0.13	5.6	3.7 (168)
5	56	Female	Colon cancer	F0	NA	NA	3.7 (270)
Chronic liver disease							
6	67	Male	NAFLD, colon cancer	F1	0.44	7.2	5.0 (137)
7	64	Female	NAFLD, colon cancer	F1	0.33	6	7.4 (270)
8	65	Male	HBV, HCC	F2	0.61	NA	4.8 (228)
9	57	Female	NAFLD, HCC	F2	0.65	12.0	7.9 (270)
10	72	Male	NAFLD, colon cancer	F2	0.77	18.0	8.0 (270)
11	45	Male	HBV, HCC, transplanted	F2	0.39	13.4	6.9 (174)
12	60	Male	HBV, HCC, transplanted	F3	0.67	NA	9.0 (270)
13	60	Male	HCV, HCC, transplanted	F3	0.60	NA	10.1 (270)
14	50	Male	Biliary cirrhosis, transplanted	F4	0.94	NA	14.2 (258)
15	58	Female	HCV, HCC, transplanted	F4	0.89	NA	14.7 (180)
16	66	Female	HCV, HCC, transplanted	F4	0.95	13.0	17.0 (164)
17	61	Female	ALD, transplanted	F4	0.98	61.5	18.1 (264)
18	63	Male	ALD, HCC, transplanted	F4	0.92	42.2	19.0 (240)
19	42	Male	HBV, HCC, transplanted	F4	0.96	NA	19.9 (270)
20	47	Male	ALD, HCC, transplanted	F4	0.98	NA	23.7 (258)

NOTE. A total of 22 consecutive patients were preincluded, because a surgical sample had been obtained with assessment of AF. Two patients were excluded because of final diagnosis of liver disease for which FibroTest had not been validated: 1 extrahepatic cholestasis (pancreatic cancer) and 1 hepatic oxalosis. The remaining 20 patients were included. For analysis of strength of concordance, 1 patient was not included because of absence of FibroTest measurement.

HCC, hepatocellular carcinoma; NA, not available.

[a]Reference value for fibrosis estimate, calculated as mean/median AF from all 5-mm samples for patient surgical biopsies.

[b]These patients had a liver tumor, without any risk of liver disease and absence of fibrosis in the nontumor liver.

Supplementary-Table-S8. Multivariate regression analysis of factors associated with menopause in UK Biobank women participants

Characteristic	Event N	OR (95% CI)[1]	p-value	q-value[2]
**Age (year)**	32778	1.60 (1.58 to 1.61)	**<0.001**	**<0.001**
**Weight (kg)**	32778	1.00 (0.99 to 1.01)	0.93	0.93
**Alcohol intake**	32778		**0.004**	**0.007**
low		—		
mild or high		0.87 (0.79 to 0.96)		
**Smoker status**	32778		**<0.001**	**<0.001**
never		—		
current		1.80 (1.53 to 2.11)		
previous		1.21 (1.10 to 1.34)		
**Body mass index (kg/m2)**	32778		0.094	0.12
<27		—		
27-<30		0.90 (0.77 to 1.06)		
30-<35		0.88 (0.74 to 1.04)		
35-<40		0.83 (0.65 to 1.06)		
40-<45		0.96 (0.68 to 1.35)		
45+		1.13 (0.70 to 1.83)		
**C-Reactive protein (mg/l)**	32778	1.02 (1.01 to 1.03)	**0.001**	**0.002**
**ALT (UI/l)**	32778	1.01 (1.00 to 1.01)	**<0.001**	**<0.001**
**AST (UI/l)**	32778	1.01 (1.00 to 1.01)	0.089	0.12
**Glucose (mmol/Ll**	32778	0.90 (0.86 to 0.93)	**<0.001**	**<0.001**
**Glycated Haemoglobin Hb1Ac (%)**	32778	1.48 (1.35 to 1.62)	**<0.001**	**<0.001**
**Platelet Count (10^9 cells/l)**	32778	1.00 (1.00 to 1.00)	**<0.001**	**<0.001**
**Creatinine (µmol/l)**	32778	1.01 (1.00 to 1.01)	**<0.001**	**0.002**
**Total Cholesterol (mmol/l)**	32778	1.23 (1.16 to 1.30)	**<0.001**	**<0.001**
**HDL Cholesterol (mmol/l)**	32778	1.83 (1.25 to 2.67)	**0.002**	**0.003**
**Apolipoprotein-A1 (g/l)**	32778	0.69 (0.44 to 1.07)	0.10	0.12
**Albumin (g/l)**	32778	1.12 (1.10 to 1.14)	**<0.001**	**<0.001**
**Total Bilirubin (µmol/l)**	32778	0.95 (0.92 to 0.99)	**0.014**	**0.022**
**Direct Bilirubin (µmol/l)**	32778	1.15 (0.93 to 1.42)	0.20	0.21
**GGT (UI/l)**	32778	1.00 (1.00 to 1.00)	0.17	0.19
**Triglycerides (mmol/l)**	32778	1.05 (0.98 to 1.13)	0.20	0.21
**Testosterone (nmol/l)**	32778	0.90 (0.86 to 0.96)	**0.001**	**0.002**

Menopause was associated with CFs related to diabetes (serum glucose and HbA1c), dyslipidemia, inflammatory markers, and ALT. The testosterone level was lower in menopausal women. Smoking status remained associated, and the average alcohol intake was still lower in menopause vs no-menopause, in line with the apoA1 decrease.

Supplementary-Figure-S1. BARICAN participants included and not included

Summary of the BARICAN (Bariatric surgery supported by ICAN consortium) study design, was fully published by Pais et al, Hepatology 2022. In comparison with the previously published article, 55 patients were included out of the original cases. Fibrosis stages (F0 to F4) were those of the CRN scoring systems.

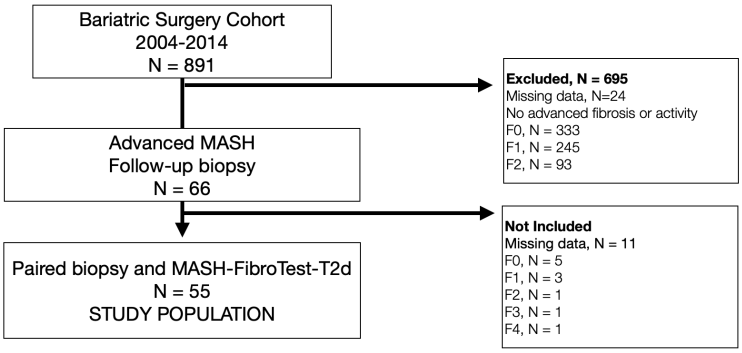

Supplementary-Figure-S2. Patients not included in the UK-Biobank general population. Among the160,380 participants at risk of MASLD pre-included, 159,794 without missing data were included in the study

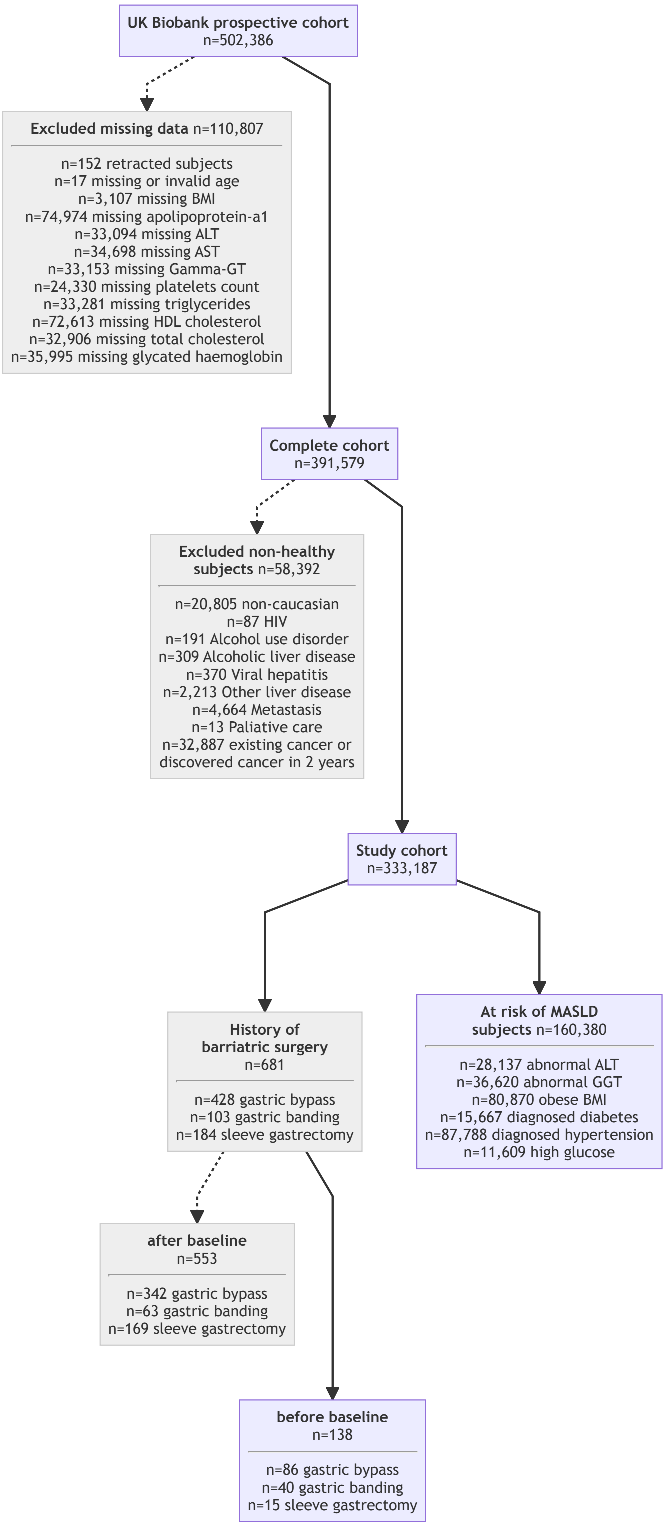

Supplementary-Figure-S3. FibroTest-T2D progression/regression rates in BARICAN participants before BS (biopsy-1), between BS and follow-up (biopsy-2), and after biopsy-2 according to age and sex. A total of 355 FibroTest-T2D were assessed, a median of four per patient.

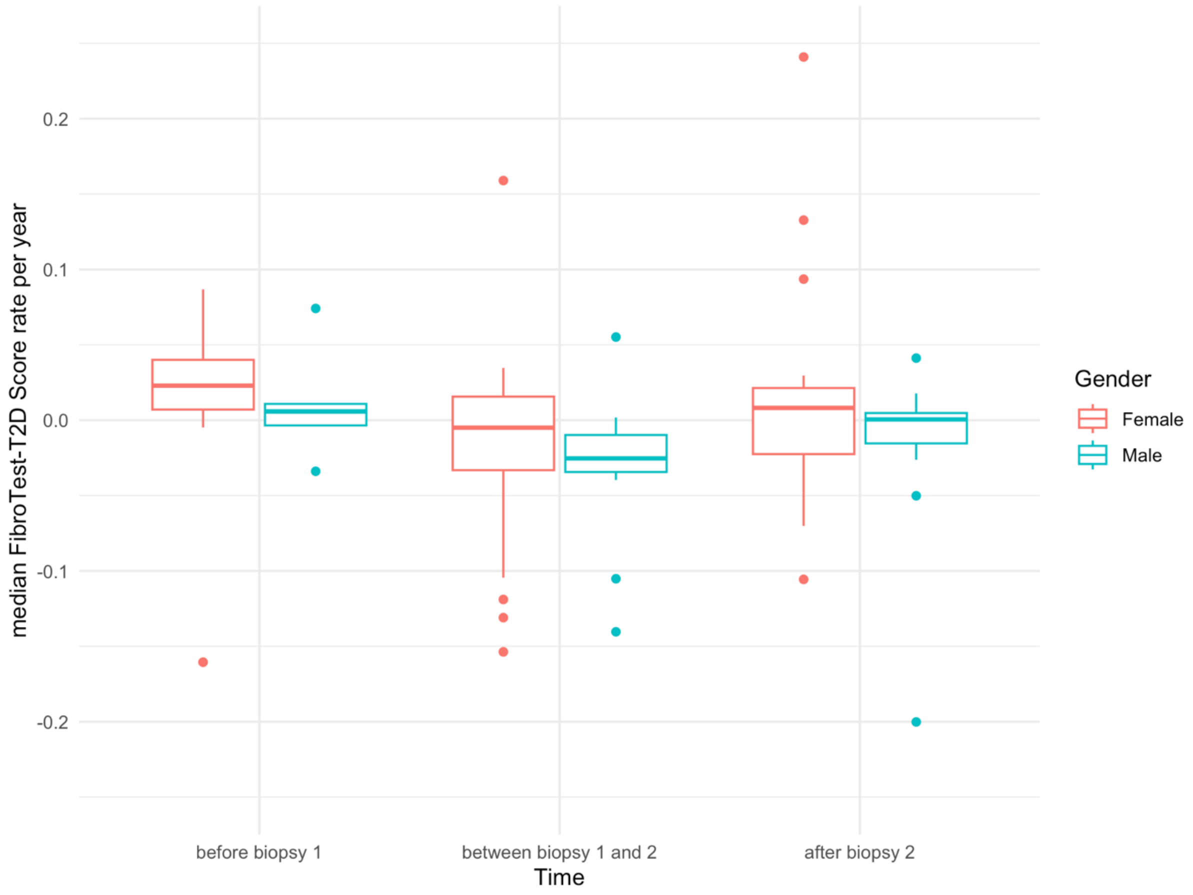

Supplementary-Figure-S4. Apolipoprotein A1 according to sex, T2D and BMI in France-FibroTest, USA-FibroTest and subsets

Supplementary-Figure-S4A. In UK-Biobank ApoA1 increased until 50 years of age in women, regardless of BMI.

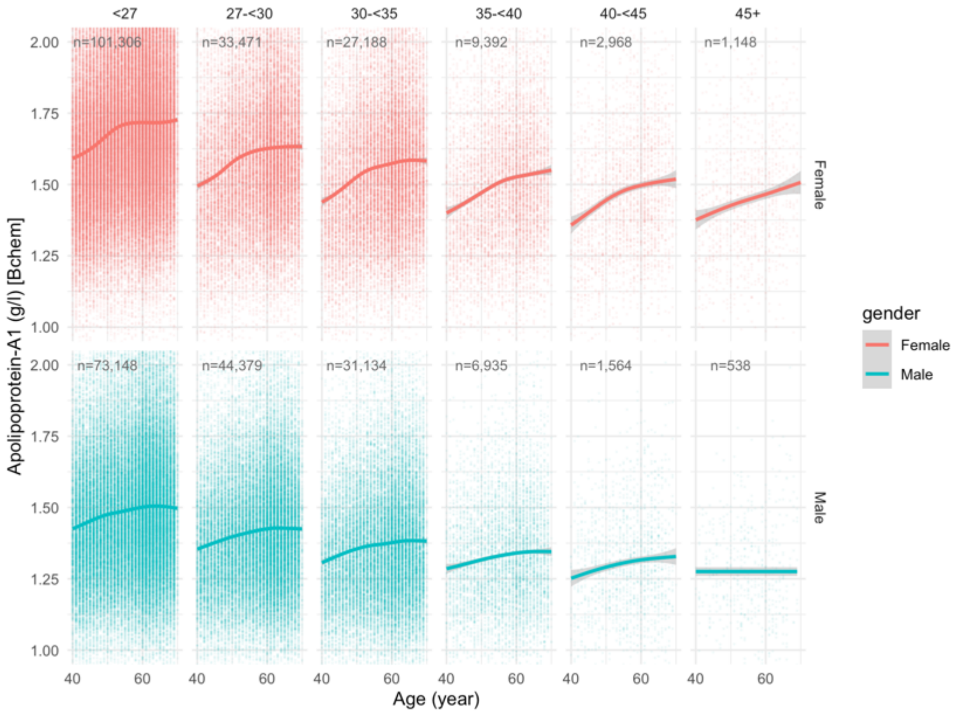

Supplementary-Figure-S4B. In France, in men with T2D and in women with a BMI of ≥27, the apoA1 increases completely disappeared.

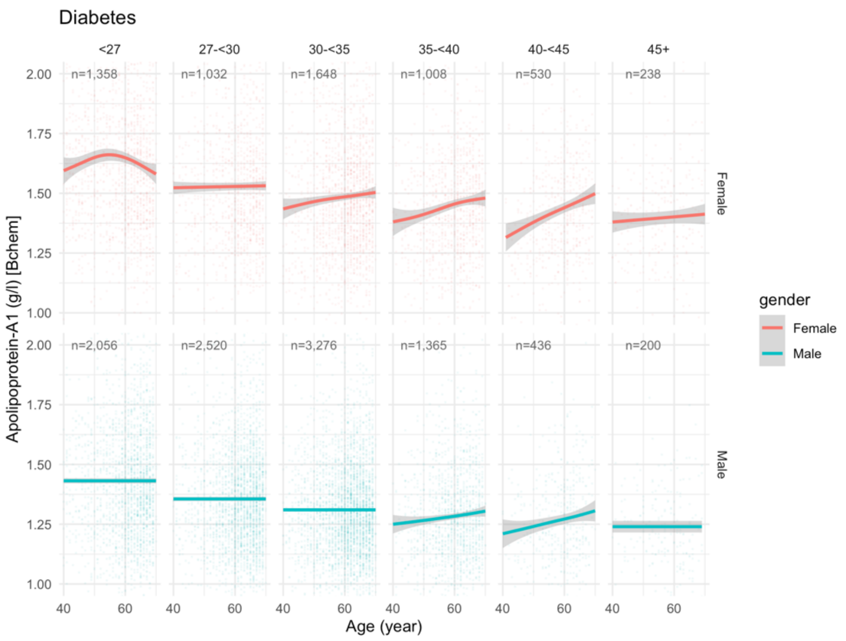

Supplementary-Figure-S4C. Using NMR, in UK-Biobank, the flat trajectory of apoA1 was confirmed in men with T2D and women with a BMI of ≥27.

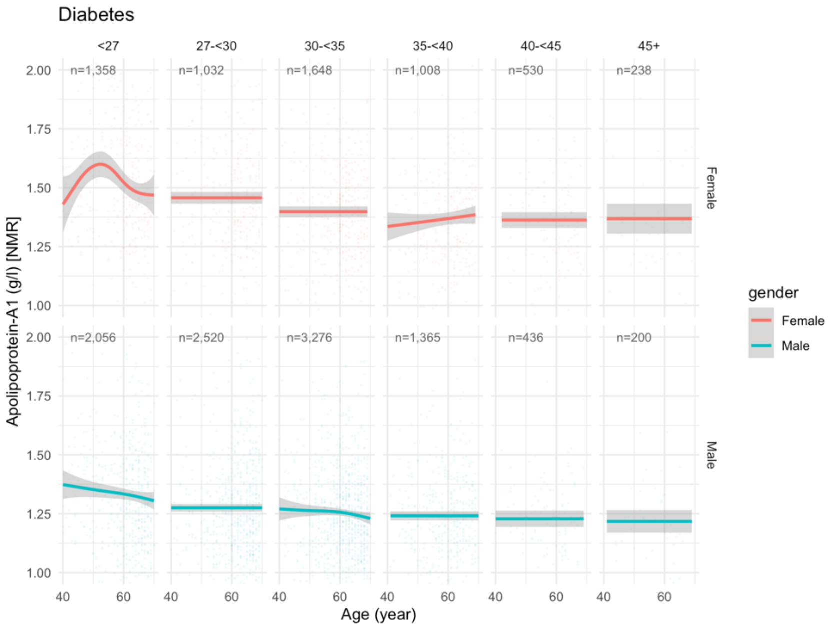

Supplementary-Figure-S4D. ApoA1 according to T2D (Glucose as proxy) in France subset

No T2D

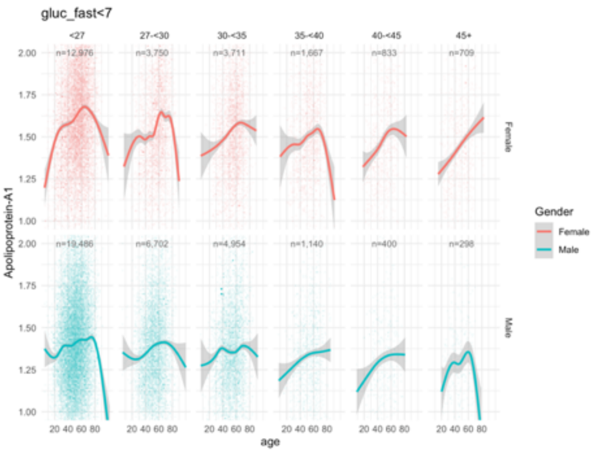

T2D

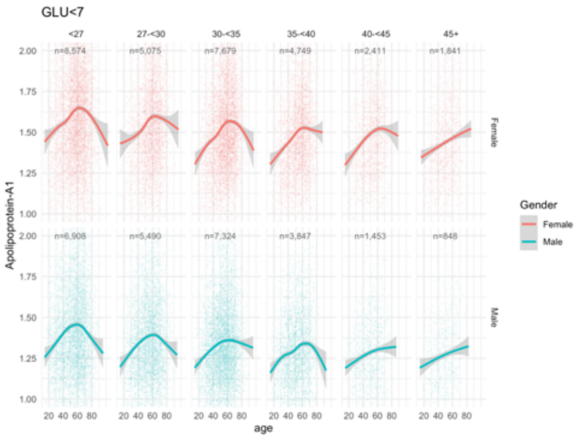

Supplementary-Figure-S4E. ApoA1 according to T2D (Glucose as proxy) in USA subset

No T2D

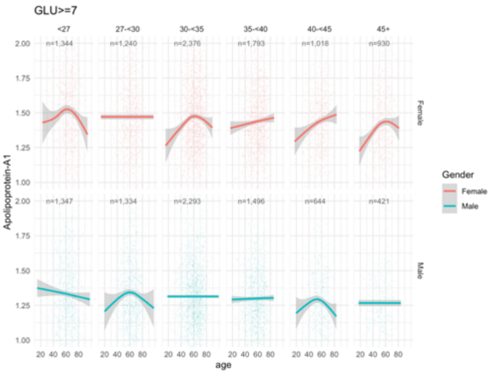

T2D

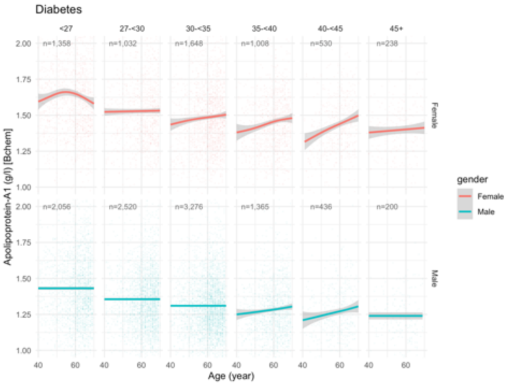

Menopause was significantly (all <.001) associated in multivariate analysis with CFs or components mainly related to diabetes (HbA1c), dyslipidemia (apoA1), inflammatory markers (C-reactive protein, low albumin), and ALT. When adjusted with all the covariables, menopause was no longer associated with BMI or hormone therapy, age remained associated and smoking status remained associated. Average alcohol intake was still lower in menopause vs no-menopause, in line with the apoA1 decrease (**Supplementary-Table-S9**).

In these large MASLD-risk populations CRN-5tier stages F1F2 were more frequent than in populations with other liver disease.

Supplementary-Figure-S5. Haptoglobin in France-FibroTest and USA-FibroTest subset according to sex, T2D (glucose >=7 as proxy) and BMI.

Regardless of age and the absence (**Supplementary-Figure-S5A and S5B)** or presence of T2D (**Supplementary-Figure-S5C and S5D**), the mean haptoglobin was always higher in women with a BMI greater than 27 kg/m^2 with a decrease according to age, in comparison with men.

5A. No T2D, France **5B. No T2D, USA**

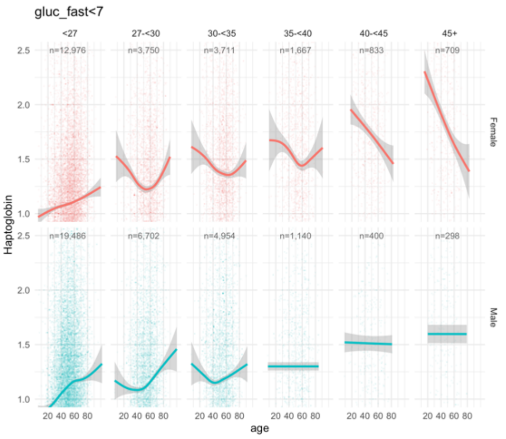

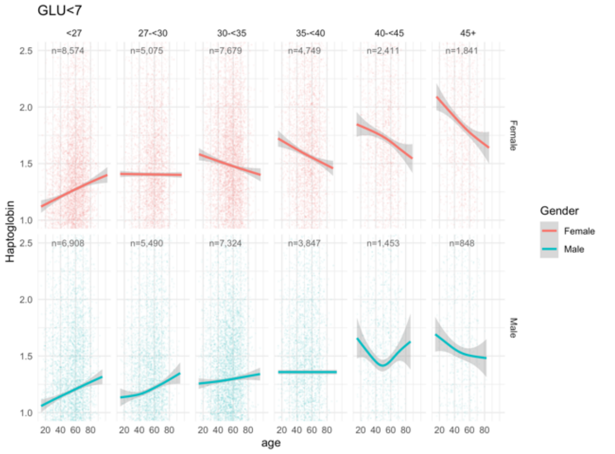

5C. T2D, France **5D. T2D, USA**

France **USA**

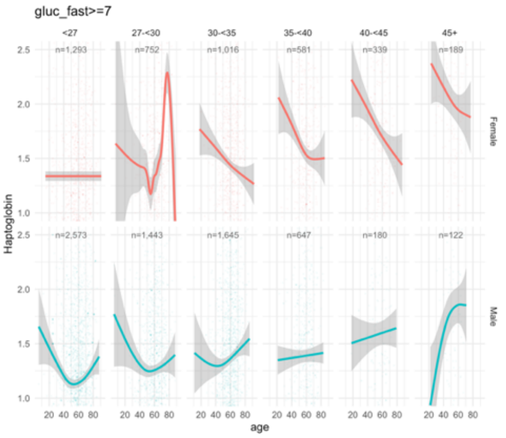

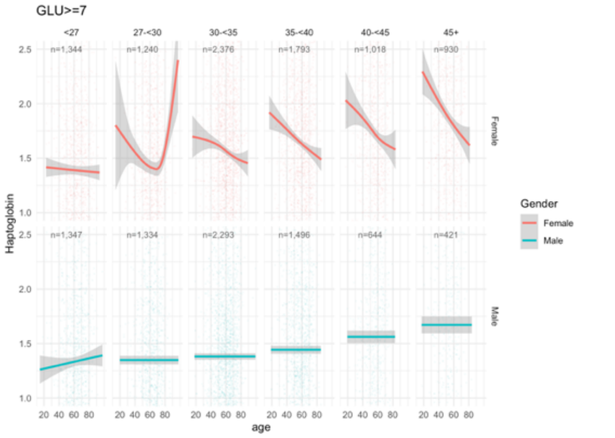

Supplementary-Figure-S6. Alpha-2 Macroglobulin in France-FibroTest vs USA-FibroTest subset according to sex, T2D (Glucose as proxy) and BMI

Mean A2M had a U shape both in women and men (**Supplementary-Figure-S6A and -S6B)**, but the increase in men started 10 years earlier than in women, especially among men with a BMI >30 kg/m^2. In men with T2D, this U shape mostly disappeared (**Supplementary-Figure-S6C)**.

6A. No T2D, France

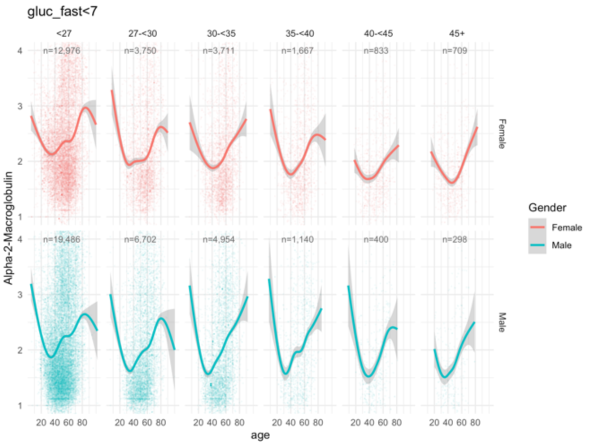

6B. No T2D, USA

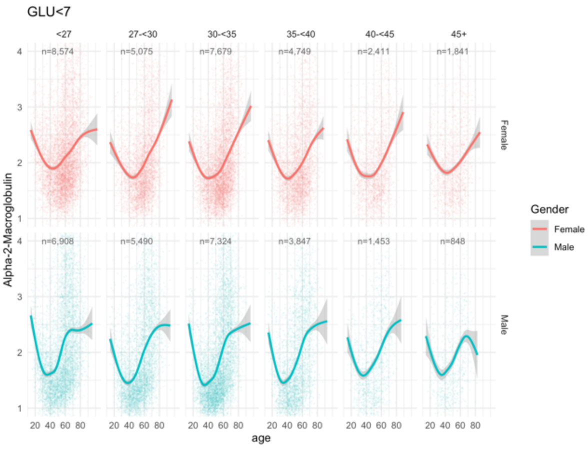

6C. T2D, France

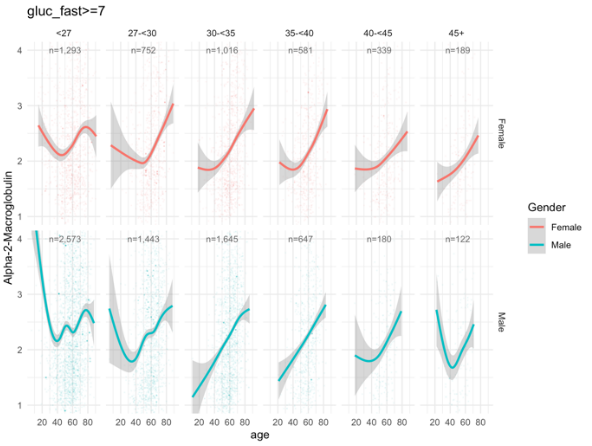

6D. T2D, USA

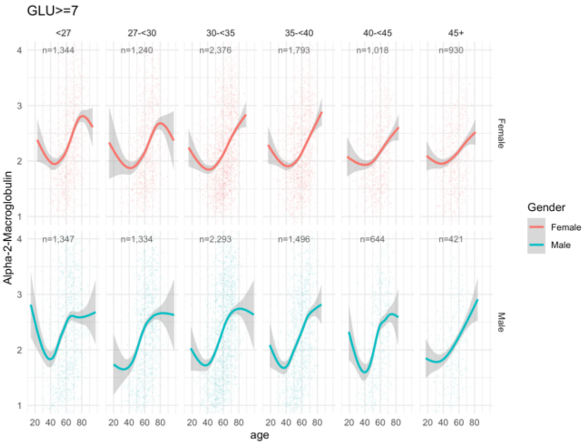

Supplementary-Figure-S7. Men Fibrosis progression rates in USA and France populations at risk of MASLD

F1 is the first stage, F2 the second and F3 (early bridging) the third stage of a fibrosis 7-tier grading scale, measured by the FibroTest-T2D-proxy.
The horizontal axis is the age. The vertical axis is the cumulative fibrosis rate assessed by the cumulative hazard-ratio. The lines are the trajectory with 95% limits, according to the presence (+) or absence of confounding main factors besides aging: black line is Glu+Ob+ =type-2 diabetes + estimated by fasting glucose >=7 and BMI >27 kg/m^2 (Ob+), red line is Glu+Ob-, blue line is Glu-Ob+ , and green line is Glu-Ob-.

F1 **F2** **F3**

Men USA-FibroTest

Panel A

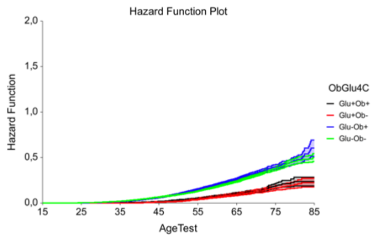

Panel B

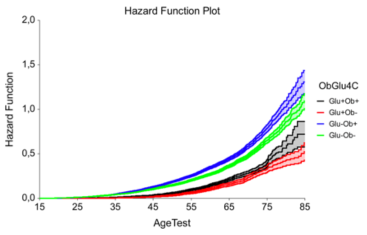

Panel C

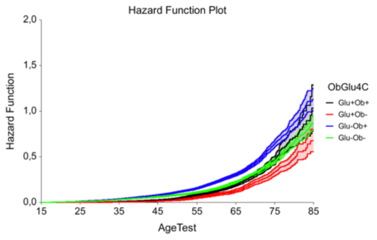

Men France-FibroTest

Panel D

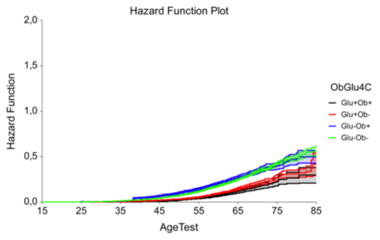

Panel E

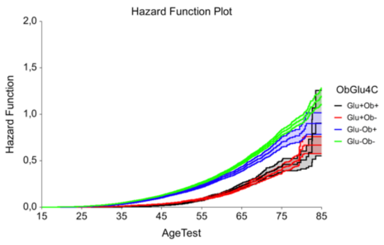

Panel F

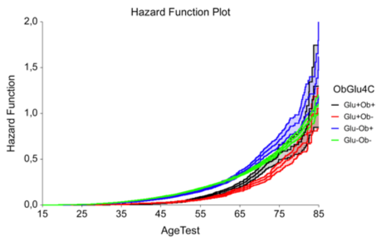

SUPPLEMENTARY-FILES

Supplementary-File-S1. Accuracy and uncertainty definitions.

STARD statements

The Standards for Reporting Diagnostic Accuracy (STARD) statement was first published in 2003 and updated in 2015 with a list of 30 essential items that should be included in every report of a diagnostic accuracy study. This update incorporates recent evidence about sources of bias and variability in diagnostic accuracy and is intended to facilitate the use of STARD. As such, STARD 2015 may help to improve completeness and transparency in reporting of diagnostic accuracy studies.[1]

Uncertainty is cited in a single statement but shortly in the revised criterion #15:

In the 2015 revised version **uncertainty** was only presented once in the following paragraph:

"Having a general list may necessitate additional instructions for informative reporting, with more information for specific types of tests, specific applications, or specific forms of analysis. Such guidance could describe the preferred methods for studying **and reporting measurement uncertainty**, for example, without changing any of the other STARD items. The STARD group welcomes the development of such STARD extensions and invites interested groups to contact the STARD executive committee before developing them."

The 3-methods (3M) appeared briefly and for the first time in the revised version **in criterion #15**: "How indeterminate index standard results **or comparator** were handled".[1]

This sentence is not clear enough for a clinician. The handling of the index result (i.e. a fibrosis NIT) is completely different than the handling of the comparator (i.e. the granularity of an histological scoring system or the variability associated with the biopsy length).

The unique STARD statement possibly concerning the 3M was reported in 22% in 2019, without details concerning the report of the comparator uncertainty

This criterion #15 was only reported in 28% of the diagnostic accuracy articles published in the journal 'Radiology' in 2016 but still under reported, 22% in 2019, contrarily to the other STARD criteria mostly improved. No such overview was published for circulating markers[2]

It is disappointing as since 2015, a specific reporting for the diagnostic accuracy of liver fibrosis marker (FIBROSTARD) was available.[3] It was derived from STARD with even more recommendations to quantify uncertainty (i.e. the **criterion #13**). So far only 12 publications have cited FIBROSTARD between 2015 and 2024,

An extension of STARD for reporting on liver fibrosis tests (Liver-FibroSTARD) has recommended methods in criterion #13.7, "the methods useful for the control of spectrum effect such as Obuchowski method,[3] and DANA score,"[4]

Indeed, uncertainty was still not identified and not discussed in a recent review of liver biopsy limitations in MASLD.[22]

Forthcoming revised version should recommend essential items for uncertainty

Uncertainty of the NITs' histological comparator is less familiar than the uncertainty of NITs. Accuracy and uncertainty should be separately explained in forthcoming STARD and FibroStard statements.

In liver fibrosis, the length of the comparator (liver biopsy) is the main responsible of the overall uncertainty as even a perfect NITs cannot reach the 0.80 binary AUROC cutoff. However, it is commonly assumed that a small amount of uncertainty in the

comparator's classifications will negligibly affect the measured performance of a diagnostic test.[2] Indeed, uncertainty was still not identified and not discussed in a recent review of liver biopsy limitations in MASLD.

Differences between Accuracy and uncertainty

In general use, the words accuracy and uncertainty describe how sure we are of something. However statistical diagnosis-methods used another definition, accuracy being the "percent of patients correctly classified as true-positive and true-negative" for the validation of fibrosis-NITs. This is not the internationally definition: "the closeness of the agreement between the result of a measurement and a true value of the thing being measured", and the definition adds that "accuracy is a qualitative concept expressed as being high or low, but not with numbers"(**Supplementary-File-S1**). In the absence of a perfect comparator with the appropriate granularity, even with a perfect fibrosis NIT and ideal biopsies, a 90% of correct-classification cannot be reached.[10-15]

Uncertainty of measurement acknowledges that no measurements can be perfect and is defined as a "parameter, associated with the result of a measurement, that characterizes the dispersion of values that could reasonably be attributed to the thing being measured". Therefore, any comparison between these NITs must also account for their comparator uncertainty, the benefit-risk, as well as the major confounding factors (CFs) and the context of use.

1. Bossuyt PM, Reitsma JB, Bruns DE et al (2015) STARD 2015: an updated list of essential items for reporting diagnostic accuracy studies. BMJ Br Med J. https://doi.org/10.1373/clinchem.2015.246280
2. McGenity C, Bossuyt P, Treanor D. Reporting of Artificial Intelligence Diagnostic Accuracy Studies in Pathology Abstracts: Compliance with STARD for Abstracts Guidelines. *J Pathol Inform*. 2022;13:100091. Published 2022 Feb 18. doi:10.1016/j.jpi.2022.100091
3. Boursier J, de Ledinghen V, Poynard T, et al. An extension of STARD statements for reporting diagnostic accuracy studies on liver fibrosis tests: the Liver-FibroSTARD standards. *J Hepatol*. 2015;62(4):807-815. doi:10.1016/j.jhep.2014.10.042
4. Poynard T, Halfon P, Castera L, et al. Standardization of ROC curve areas for diagnostic evaluation of liver fibrosis markers based on prevalences of fibrosis stages. *Clin Chem*. 2007;53(9):1615-1622. doi:10.1373/clinchem.2007.085795
5. Husby S, Choung RS, Crawley C, Lillevang ST, Murray JA. Laboratory Testing for Celiac Disease: Clinical and Methodological Considerations. *Clin Chem*. Published online August 5, 2024. doi:10.1093/clinchem/hvae098
6. Tarsney C. Moral Decision-Making Under Uncertainty. Stanford encyclopedia of philosophy 2024.
7. Bell S. NPL. National Physical Laboratory. Good Practice Guide No.11.The Beginner's Guide to Uncertainty of Measurement 1999
8. Is there a difference between measurement 'accuracy' and 'uncertainty'?
9. Antonelli, Giorgia, Padoan, Andrea, Aita, Ada, Sciacovelli, Laura and Plebani, Mario. "Verification of examination procedures in clinical laboratory for imprecision, trueness and diagnostic accuracy according to ISO 15189:2012: a pragmatic approach" *Clinical Chemistry and Laboratory Medicine (CCLM)*, vol. 55, no. 10, 2017, pp. 1501-1508. https://doi-org.proxy.insermbiblio.inist.fr/10.1515/cclm-2016-0894

Supplementary-File-S2. Ethics.

For Fibrosis-TAGS, large surgical samples of resected livers from 20 consecutive patients with chronic liver diseases or normal liver surrounding tumors were prospectively included (**Supplementary Table S1**). These patients must have accepted and signed a consent form and undergone hepatectomy or liver transplantation. We combined new individual data with a previous, integrated database where informed consent was obtained for all patients.[25]

For QuidNash, the study (NCT03634098) was approved by the Research Ethics Committee (#18.021-2018-A00311-54). All patients provided written informed consent. All authors had access to the study data and reviewed and approved the final manuscript.[26,34] For BARICAN, ethical approval was obtained from the Ethics Committee of Ile de France, CPP Paris VI, 1N°13577. All patients signed an informed consent form.[6]

For UK-BioBank, we performed a retrospective analysis of the prospective UK Biobank cohort (registration number UKB-ID 670334).[37]

USA-FibroTest and France-FibroTest are non-interventional studies and were exempt from institutional review board (IRB) review (ethics committee of "Comité de Protection des Personnes of Paris, Ile-de-France" FIBROFRANCE project; CPP-IDF-VI, 10–1996-DR-964, DR-2012-222, and USA-NCT01927133). No patient consent was required, as all data were analyzed anonymously.[38,39]

The LIDO study group is a subset of the FibroFrance project centralized in the Pitié-Salpêtrière Hospital, Paris, France (USA-NCT01927133). After providing written informed consent, every patient underwent a liver biopsy, during which two liver samples were extracted.[21]

The inclusion criterion was a FibroTest prescription for each subject through an authorized laboratory connected to the BioPredictive systems. Exclusion criteria were possible duplicates (same date of birth, sample date, country, and gender).

Supplementary-File-S3. BARICAN subset design for validating bariatrics surgery (BS).

In the present paper we presented the CRN detailed scoring system of Kleiner et al (Gastroenterology 2005) revised by Harrison et al (NEJM 2024) including the sub-stage F1B as a new F1 stage, F1BA and F1B considered as low fibrosis being classified in stage F0 with cases without fibrosis.
We detailed in this supplementary file a third score the Elucidating Pathways of Steatohepatitis (EPOS), a nine-tier fibrosis score including the early stages of early fibrosis (eF), similar to CRN-F1B, but not using a F0 score including F0, F1A and F1C).

BARICAN subset design (Pais et al, Hepatology 2021).
Barican Patients
This retrospective analysis of patients who underwent bariatric surgery and were prospectively recruited in the Nutrition Department from Hôtel-Dieu and Pitié-Salpêtrière hospitals from 2004 to 2014. All patients were ≥18 years old and met the criteria for bariatric surgery, that is, body mass index (BMI) ≥40 kg/m2 or BMI ≥35 kg/m2 and at least one obesity-related comorbidity among the following: high blood pressure, type 2 diabetes, dyslipidemia, or obstructive sleep apnea (OSA).To determine the histological benefit of bariatric surgery, patients with significant liver injury and with a stable weight for at least 6 months were proposed to have a follow-up liver biopsy (LB) ≥1 year after surgery. Significant liver injury was defined as either: (1) AF (i.e., BF or cirrhosis NASH Clinical Research Network stages 3 or 4, respectively), regardless of the activity grade, or (2) high activity (HA) grade (Steatosis, Activity, and Fibrosis [SAF] activity score A3 or A4; see below), regardless of the fibrosis stage, both documented during the perioperative LB. Exclusion criteria were: a follow-up after bariatric surgery shorter than 1 year, surgical procedures other than sleeve gastrectomy or Roux-en-Y gastric bypass, inflammatory or infectious diseases, neoplasia, and other causes of chronic liver disease (viral hepatitis B or C, autoimmune liver disease, hemochromatosis, or alcohol consumption of ≥20 g/d in women and >30 g/d in men).

Barican Clinical and laboratory assessment
Clinical data were recorded at baseline and 1 and 5 years after bariatric surgery and at the time of the follow- up liver biopsy. Weight at 1 and 5 years after bariatric surgery and at the follow-up biopsy was recorded. Body composition was assessed at baseline using whole-body fan-beam dual-emission x-ray absorptiometry scanning. T2D was defined according to the American Diabetes Association criteria as follows: fasting plasma glucose (FPG) ≥7 mmol/L (126 mg/dL) and/or 2-h plasma glucose ≥11 mmol/L (200 mg/dL) and/or hemoglobin A1c (HbA1c) ≥6.5% and/or the use of any glucose-lowering drugs. Baseline information was collected on diabetes duration before surgery. At follow-up, diabetes remission was defined as partial remission (if HbA1c < 6.5%, FPG between 5.6 and 6.9 mmol/L, and no use of glucose-lowering drugs) or complete remission (if HbA1C < 6%, FPG < 5.6 mmol/L, and no use of glucose-lowering drugs).
High blood pressure was defined as systolic blood pressure ≥130 mm Hg or diastolic blood pressure ≥85 mm Hg or the use of antihypertensive drugs; resolution of hypertension was defined as normal blood pressure in the absence of antihypertensive treatment. Dyslipidemia was defined as LDL ≥4.14 mmol/L or specific treatment for lipid disorders or HDL < 1.03 mmol/L in males or <1.29 mmol/L in females; resolution of dyslipidemia was defined as normal LDL and HDL values in the absence of lipid-lowering therapy. Fasting blood samples were collected at baseline, 1 year, and the end of follow up to measure fasting glucose, fasting insulin, HbA1c, lipid profile, and liver function tests.

Barican Liver histology

Surgical LBs were systematically performed in all patients during the bariatric surgery procedure. Liver tissue samples were stained with hematoxylin and eosin and picrosirius red.

Follow-up LBs were performed transcutaneously using a 16-G needle, or, if contraindicated, by transjugular route. Both baseline and follow-up LBs (glass slides) were reviewed by a single pathologist and scored using the SAF classification.

The SAF activity score was calculated by adding the grades of hepatocellular ballooning (0-2) and lobular inflammation (0-2). Liver fibrosis was semi quantitatively assessed using the standard-CRN staging system: F0, normal; F1, focal pericellular fibrosis in zone 3; F2, perivenular and -cellular fibrosis confined to zones 2 and 3, with or without portal or periportal fibrosis; F3, BF or extensive fibrosis and no clear-cut cirrhosis; and F4, cirrhosis.

To provide more granularity for AF, and in particular for BF stages, we further used the new EPoS seven tier classification as follows: F0, normal; F1: NASH-CRN stages 1a, 1b, and 1c; F2, central or portal fibrosis + lobular fibrosis or portal + central fibrosis; F3, bridging with few septa (≤2/10-mm biopsy length); F4, bridging with numerous septa (>2/10-mm biopsy length); F5, many septa with few nodules (incomplete cirrhosis with nascent regenerative nodules); and F6, annular fibrosis with complete nodulation. Histological outcomes measured at follow- up biopsy were: (1) fibrosis response defined as the absence of BF at follow-up (i.e., stages 0, 1, or 2 only); (2) activity response defined as a low activity grade (A0, A1, or A2); (3) resolution of NASH defined as clearance of ballooning together with clearance of lobular inflammation or persistence of only mild inflammation (grade 1); and (4) presence of normal liver: no steatosis, no activity with no ballooning, and no or minimal inflammation and the absence of fibrosis (S0A0/A1F0).

In comparison with participants at risk of MASLD, those with BS were significantly younger, had lower alcohol consumption, higher serum HbA1c, glucose, C-reactive protein, and platelet counts but lower transaminases, FIB4, creatinine, apoA1, and total cholesterol.

Supplementary-File-S4. Summary of QuidNash protocol for validating FT-T2D

Research design and methods

The primary outcome of this prospective cross-sectional multicentre study in patients with type 2 diabetes was to assess the diagnostic accuracy of the FibroTest, NashTest-2 and SteatoTest-2 using liver histology as the reference to evaluate liver fibrosis, activity and steatosis. MASLD was suspected based on the presence of abnormal liver enzymes as well as an ultrasound scan showing a bright liver echo pattern, in patients with type 2 diabetes diagnosed in a diabetology outpatient clinic. Consecutive patients were prospectively recruited between October 2018 and 2020 in four outpatient diabetology clinics in the Assistance-Publique-Hôpitaux-de-Paris (File S2).

Main analyses

The primary objective of the study was to evaluate the diagnostic accuracy of each component of the NashFibroTest, the FibroTest, the NashTest and the SteatoTest, in relation to the histological evaluation of fibrosis, Nash activity and steatosis. The primary endpoint was the validation of the FibroTest because the stage of fibrosis is the main prognostic criterion compared the grades of Nash or Steatosis.

Inclusion and exclusion criteria in the validation population

Inclusion criteria were as follows: patients were ≥18 years of age, able to give written informed consent, with type 2 diabetes defined according to American Diabetes Association or World Health Organization criteria, and were scheduled, independently from this study, to undergo a liver biopsy for investigation of suspected NAFLD within 4 weeks after ultrasonography and alanine aminotransferase (ALT) assessment. These patients had abnormal transaminases had to be negative for standard tests for liver diseases. Exclusion criteria were as follows: patients with HBV, HCV and autoimmune diseases, pregnant women, patients without national health insurance, with a history of chronic liver disease, patients with serum hemoglobin <7g/L or <10g/L in the presence of cardiovascular or pulmonary disease, patients who refused liver biopsy or tests, patients with significant alcohol consumption (≥30 g/ day for males and ≥20 g/day for females) and by serum carbohydrate deficient transferrin per cent >2%, and patients with a terminal disease.

Histopathologic evaluation in the validation group

Liver biopsy (intercostal or transvenous) was performed in all patients according to the standard local procedure. Biopsy specimens were fixed in formalin, embedded in paraffin and stained with hematoxylin and eosin and Sirius Red. Slides were analyzed in each center by an experienced pathologist and then centrally reviewed by a single experienced pathologist (PB) for the read-outs, blinded to all patient characteristics. The length and the number of fragments were assessed, and the quality scored according to a three-class classification (adequate, marginal and inadequate). The cause of any inadequate liver biopsy was specified: length, fragmentation or technical issues, that is, inadequate staining, or granuloma. MASH was diagnosed according to the presence of steatosis, hepatocyte ballooning (three grades 0-2) and lobular inflammation (three grades 0-2) with at least 1 point for each category. MASLD activity (Nash score) was scored using both the SAF (main outcome in four classes),8-10 and NASH-CRN scoring systems, which are different for several feature scores. Fibrosis was scored using the same SAF and CRN definition in five stages from 0 to 4.21 Steatosis was scored in four grades (from 0, 'less than 5%', 1 '5%-30%', 2 '33%-66%', to 3 'more than 66% of hepatocyte with steatosis'). Portal inflammation and Mallory bodies were also recorded by grade into three classes.

Supplementary-File-S5. 3-Methods methodology details and examples

DANA (Difference between Advanced and Non-Advanced fibrosis) or Spectrum-Index, to predict an adjusted-AUROC for F3F4 5-tier diagnosis

Ninety percent of studies designed to validate noninvasive tests for the diagnosis of metabolic liver disease continue to use the standard-AUROC as a summary measure of diagnostic accuracy, even if the risk of spectrum effect and the type-one error of the tests comparing these measures in two samples with different distributions of stages or grades, have been identified.

In the absence of wAUROC, we previously constructed an index [DANA or index of fibrosis spectrum variability (Spectrum-Index)] in patients at risk of MASLD,[1] to predict an adjusted-AUROC for F3F4 diagnosis from the prevalences of biopsy proven fibrosis stages, as published for the first time in patients with chronic hepatitis C.[2]
This index did not need the individual data, was assessed in all the possible combinations of fibrosis stages using data-base with and without patients with T2-diabetes. This index permitted to assess the variability of the standard (binary) bAUROCs for the diagnosis of fibrosis stages F3F4 of three biomarkers, FibroTest, transient elastography and magnetic resonance elastography, due to the presence of T2-diabetes, after adjustment according to the prevalences of fibrosis stages, from an-overview of 25 published studies.

This is possible when the standard bAUROC is known as well as the number of patients by class of fibrosis stages.

Diagnostic performances of FibroTest in T2-diabetes vs. controls: wAUROC (Obuchowski measures) in 600 patients at risk of MASLD and DANA or index of fibrosis spectrum variability (Spectrum-Index)

Regression curves permitted to calculate an adjusted DANA according to the stages spectrum and the adjusted F34-adjAUROC. Firstly an adjusted-DANAF34 is constructed: 3,06952 + 7,12510*bAUROCF34 and then the adjusted-bAUROCF34 = 0,5035+0,1059*adjusted-DANAF34.[1]

According to the histological endpoint, the binary AUROCs of blood test varied in patients with T2-diabetes and suspected NASH from 0.575 to 0.801, without any significant differences with the performances in matched controls, which also varied from 0.555 to 0.848
According to the histological endpoint, the wAUROCs of blood test varied in patients with T2-diabetes and suspected NASH from 0.575 to 0.801, without any significant differences with the performances in matched controls, which also varied from 0.555 to 0.848.

Variability of the area under the receiver operating characteristic curves in the diagnostic evaluation of liver fibrosis markers: impact of biopsy length and fragmentation.

Binary-AUROC increased the uncertainty of the indirect comparisons and weighted-AUROCs must be used as recommended

The standard binary-AUROC for the diagnosis of significant liver fibrosis can vary according to the prevalence of each stage, and a binary-AUROC for advanced fibrosis (i.e. F2

F3 F4 vs F0 F1) of the same test in the same disease has varied from 0.67 to 0.98.[5] In patients with type 2 diabetes, the influence of the spectrum effect on bAUROCs explains the misleading interpretation of bAUROCs in the absence of face-to-face comparisons between NITs.[7,9]

It is commonly assumed that a small amount of uncertainty in the comparator's classifications will negligibly affect the measured performance of a diagnostic test. This not true. A 5% or greater misclassification rate by the comparator can lead to significant underestimation of true test performance. An online simulation tool allows researchers to explore this effect using their own trial parameters (https://imperfect-gold-standard.shinyapps.io/classification-noise/) and the source code is freely available (https://github.com/ksny/Imperfect-Gold-Standard).

Uncertainty and the impact of biopsy length

Doubling the length of the median biopsy from 20 mm to 40 mm increased the prevalence of bridging fibrosis CRN stage F3 from 25% to 33%, that is, it reduced the uncertainty (misclassification) of 8% when staging 20 mm biopsies in MASLD.[21] Used large surgical samples of resected livers from patients we assessed the strength of the correlation between FibroTest and the area of fibrosis of 27,869 virtual biopsies in 6,500 patients. The correlation increased from 0.685 to 0.777 between 5 mm and 30 mm (P<.0001).[25]
Here, the wAUROCs were stratified using the median of biopsy length as a cutoff to prevent an a priori bias of arbitrary length. The medians were 17 mm in the QuidNash study[26,35] and 20 mm in the BARICAN study.[6]

Mathematically, the uncertainty of a comparator decreases the accuracy of any circulating test and increases the uncertainty of fibrosis stages' prevalence estimates, inducing a spectrum bias and therefore decreasing the predictive values.[18] Using a true reference with large surgical biopsies, we previously assessed this uncertainty in chronic liver disease.[25] The worse performances (wAUROC) between adjacent CRN 5-tier stages, even for a 30 mm biopsy length, were previously observed between F2 and F1 (0.505) and between F2 and F3 (0.552), the zone F2–F3 of early bridging in fibrosis area.[22] Here, the impact of biopsy length was originally assessed using the QuidNash and BARICAN studies (**Table 2**).

We demonstrated that the overall uncertainty of biopsy assessed for the first time by wAUROC increased with biopsy length from 0.885 for 5-mm to 0.912 for 30-mm samples ($P<0.0001$). More recently we assessed prospectively the effect of biopsy uncertainty in 272 T2D patients with a median 17 mm long biopsy, using a published online simulation tool and our trial parameters.[8] The expected bAUROC of the FibroTest cannot be more than 0.70 whatever its real performance due to the 30% misclassification rate of the 17mm biopsy as comparator. This simple result is a major warning for all qualification of NITs without assessing the comparator uncertainty. From these evidence-based results, only NITs using biopsies with length greater or equal to 30 mm can pretend to an uncertainty lower than 20% for predicting bridging progression or regression.
The NIMBLE report does not contain any sensitivity analysis on the impact of biopsy length on the binary AUROCs of the pre-qualified or qualified tests.[1] In the digital pathology review two recommendations were cited: "Using a 16-gauge biopsy needle with at least a 20mm core is generally considered a 'best practice' for assessing MASLD" and "Sample size calculations suggested that a biopsy sample that was 22 mm in length was sufficient for a good estimation of collagen proportionate area (CPA), but stage classification is non-linear and required more tissue."

We estimate the FibroTest uncertainty related to the biopsy sample length. performed in various liver diseases (including 909 MASLD cases). [9] We analyzed digitized images of 27,869 virtual biopsies of increasing length as well as data from 6,500 patients with interpretable FibroTest results who also underwent biopsy analysis. We used the CRN 5-tier scoring system and determined the fibrotic areas of large surgical samples collected from 20 consecutive patients with chronic liver disease or normal liver tissue. A total of digitized images of 27,869 virtual biopsies of increasing length and analyzed data from 6,500 patients with interpretable FibroTest results who also underwent biopsy analysis. This study challenged the misleading usual statement that there is a gray zone for biomarkers compared to biopsy.

The biopsy fibrosis scoring system is the method for control of misclassification errors by the reference test, named as the noise (classification uncertainty) of the comparator.[51] This uncertainty is directly correlated to the biopsy length.[34-37,52.]

Here we use a stratification of wAUROCs according to median biopsy length which can assess the impact of the biopsy length on the wAUROC as previously published.[36] We demonstrated that the overall uncertainty of biopsy assessed for the first time by wAUROC increased with biopsy length from 0.885 for 5-mm to 0.912 for 30-mm samples ($P<0.0001$). More recently we assessed prospectively the effect of biopsy uncertainty in 272 T2D patients with a median 17 mm long biopsy, using a published online simulation tool and our trial parameters.[33] The expected bAUROC of the FibroTest cannot be more than 0.70 whatever its real performance due to the 30% misclassification rate of the 17mm biopsy as comparator. This simple result is a major warning for all qualification of NITs without assessing the comparator uncertainty. From these evidence-based results, only NITs using biopsies with length greater than 25 mm can pretend to an uncertainty lower than 20% for predicting bridging progression or regression.

STARD essential statements are always cited in circulation biomarkers reviews but almost never applied

Reporting Diagnostic Accuracy in 2003 and updated in 2015 must be applied. After the 22 STARD essential recommendations in 2003, a 15th criterion was added in 2015: 'How indeterminate index test or reference standard results were handled'.[9] This criterion was only reported in 28% of the diagnostic accuracy articles published in the journal 'Radiology' in 2016 but without improvement, 22% in 2019, contrarily to the other STARD criteria which mostly improved. No such overview was published for circulating markers. It is disappointing as since 2015, a specific reporting for the diagnostic accuracy of liver fibrosis marker (FIBROSTARD) was available.[52] It was derived from STARD with even more recommendations to quantify uncertainty (i.e. the criterion #13). So far only 12 publications have cited FIBROSTARD between 2015 and 2024,

Finally, in the absence of these recommendations, as well as the absence of prospective validation of pre-defined cut-offs, it is difficult to know how clinicians could apply the tests since current society guidelines give no specific cut-off recommendations.

LITMUS, and NIMBLE are producing milestone results for improving the accuracy of circulating NITs in MASLD. However, our results suggest several improvements for the forthcoming versions. Both studies so far did not provide wAUROC, nor bAUROC stratification according to biopsy length, did not use EPOS fibrosis scoring system and did not

provide results in intention to diagnose. A ranking not applying the STARD recommendations related to uncertainty of the comparator, such as an arbitrary AUROC >= 80% and without head-to-head comparison is at high risk of misclassifications between NITs. We demonstrated that in intension-to-diagnose, FibroTest-T2D, VCTE, TD-SWE would not be accurate (bAUROC <0.80) but in standard analysis VCTE and TD-SWE would be accurate (bAUROC >= 0.80), without significant differences between these bAUROCs. In patients with T2D, the influence of the spectrum effect on bAUROCs explains the misleading interpretation of bAUROCs in the absence of head-to-head comparisons between NITs.

Finally, our results highlight several essential STARD recommendations that have been neglected in the past: How indeterminate results of the histological fibrosis score were handled (item # 15) which must include an improved granularity and the biopsy sample length, the (item # 14) Methods for estimating or comparing measures of diagnostic accuracy which should priories the weighted AUROC instead of the binary AUROC. For patients, knowing more about the reality of uncertainty will enable to decrease the risk of false positives or negatives associated with confounding factors.

Almost 20 years ago we stated that "many government authorities have accepted the reimbursement of health care costs of chronic liver diseases without the classical 15 mm liver biopsy sample report."[11] This permitted to treat efficiently Hepatitis C and B. As soon as the new drugs of obesity are efficient for cardiovascular mortality why do we wait to treat bridging fibrosis already identified using circulating biomarkers validated by recommended methodology ?

References for 3-Methods methodology details.

1. Poynard T, Halfon P, Castera L, et al. Standardization of ROC curve areas for diagnostic evaluation of liver fibrosis markers based on prevalences of fibrosis stages. *Clin Chem*. 2007;53(9):1615-1622. doi:10.1373/clinchem.2007.085795
2. Poynard T, Peta V, Deckmyn O, et al. Performance of liver biomarkers, in patients at risk of nonalcoholic steato-hepatitis, according to presence of type-2 diabetes. *Eur J Gastroenterol Hepatol*. 2020;32(8):998-1007. doi:10.1097/MEG.0000000000001606
3. Xiao G, Zhu S, Xiao X, Yan L, Yang J, Wu G. Comparison of laboratory tests, ultrasound, or magnetic resonance elastography to detect fibro- sis in patients with nonalcoholic fatty liver disease: a meta-analysis. Hepatology 2017; 66:1486–1501.
4. Boursier J, de Ledinghen V, Poynard T, et al. An extension of STARD statements for reporting diagnostic accuracy studies on liver fibrosis tests: the Liver-FibroSTARD standards. *J Hepatol*. 2015;62(4):807-815. doi:10.1016/j.jhep.2014.10.042
5. Guéchot J, Boursier J, de Ledinghen V, et al. Liver-FibroSTARD checklist and glossary: tools for standardized design and reporting of diagnostic accuracy studies of liver fibrosis tests. *Clin Chem Lab Med*. 2015;53(8):1135-1137. doi:10.1515/cclm-2015-0241
6. Foody GM. Challenges in the real world use of classification accuracy metrics: From recall and precision to the Matthews correlation coefficient. *PLoS One*. 2023;18(10):e0291908. Published 2023 Oct 4. doi:10.1371/journal.pone.0291908
7. Foody GM. Challenges in the real world use of classification accuracy metrics: From recall and precision to the Matthews correlation coefficient. *PLoS One*. 2023;18(10):e0291908. Published 2023 Oct 4. doi:10.1371/journal.pone.0291908
8. Poynard T, Halfon P, Castera L, et al. Variability of the area under the receiver operating characteristic curves in the diagnostic evaluation of liver fibrosis markers: impact of biopsy length and fragmentation. *Aliment Pharmacol Ther*. 2007;25(6):733-739. doi:10.1111/j.1365-2036.2007.03252.x

9. Bossuyt PM, Reitsma JB, Bruns DE et al (2015) STARD 2015: an updated list of essential items for reporting diagnostic accuracy studies. BMJ Br Med J. https://doi.org/10.1373/clinchem.2015.246280
10. Stahl A, Tietz A, Kendziora B. Has the quality of reporting improved since it became mandatory to use the Standards for Reporting Diagnostic Accuracy? Insights into Imaging 2023;14(1):85.
10. McGenity C, Bossuyt P, Treanor D. Reporting of Artificial Intelligence Diagnostic Accuracy Studies in Pathology Abstracts: Compliance with STARD for Abstracts Guidelines. *J Pathol Inform*. 2022;13:100091. Published 2022 Feb 18. doi:10.1016/j.jpi.2022.100091
11. Poynard T, Ratziu V, Benhamou Y, Thabut D, Moussalli J. Biomarkers as a first-line estimate of injury in chronic liver diseases: time for a moratorium on liver biopsy?. *Gastroenterology*. 2005;128(4):1146-1148. doi:10.1053/j.gastro.2005.02.056

Supplementary-File-S6. File Bland-Altman plot (BA) and Limits of Agreement (LOA) between NITs validated by biopsies and proxies combining components available in large populations for assessing fibrosis stages, steatosis and inflammation (MASH) grades.

Panel A. Bland-Altman limits of agreement (BA-LoA).
Abu-Arafeh A, Jordan H, Drummond, G. Reporting of method comparison studies: A review of advice, an assessment of current practice, and specific suggestions for future reports. Br J Anaesth. 2016, 117, 569–575.

**Reporting Item**	**Responses**
(1) Pre-established acceptable limit of agreement	Post-hoc proof of concept, needing validation
(2) Description of the data structure (e.g., no. of raters, replicates, block design)	No replicate
(3) Estimation of repeatability of measurements if possible (mean of differences between replicates and respective standard deviations)	No replicate
(4) Plot of the data, and visual inspection for normality, absence of trend, and constant variance across the measurement range (e.g., histogram, scatter plot)	Done; trends and not normal distributions but mostly at the extremes
(5) Transformation of the data (e.g., ratio, log) according to 4), if necessary	No transformation
(6) Plotting and numerically reporting the mean of the differences (bias)	Done differences were always inside the 95% interval
(7) Estimation of the precision, i.e., standard deviation of the differences or 95% confidence interval for the mean difference	Done
(8) Plotting and numerically reporting the BA-LoA	Done
(9) Estimation of the precision of the BA-LoA by means of 95% confidence intervals	Done
10) Indication of whether the measurement range is sufficiently wide (Preiss-Fisher procedure)	No replicate
(11) Between- and within-subject variance or stating that the confidence intervals of the BA-LoA were derived by taking the data structure into account	Not done
(12) Software package or computing processes used	NCSS software BA-LoA
(13) Distributional assumptions made (e.g., normal distribution of the differences)	Done

Panel B. Women SteatoTest NITs.

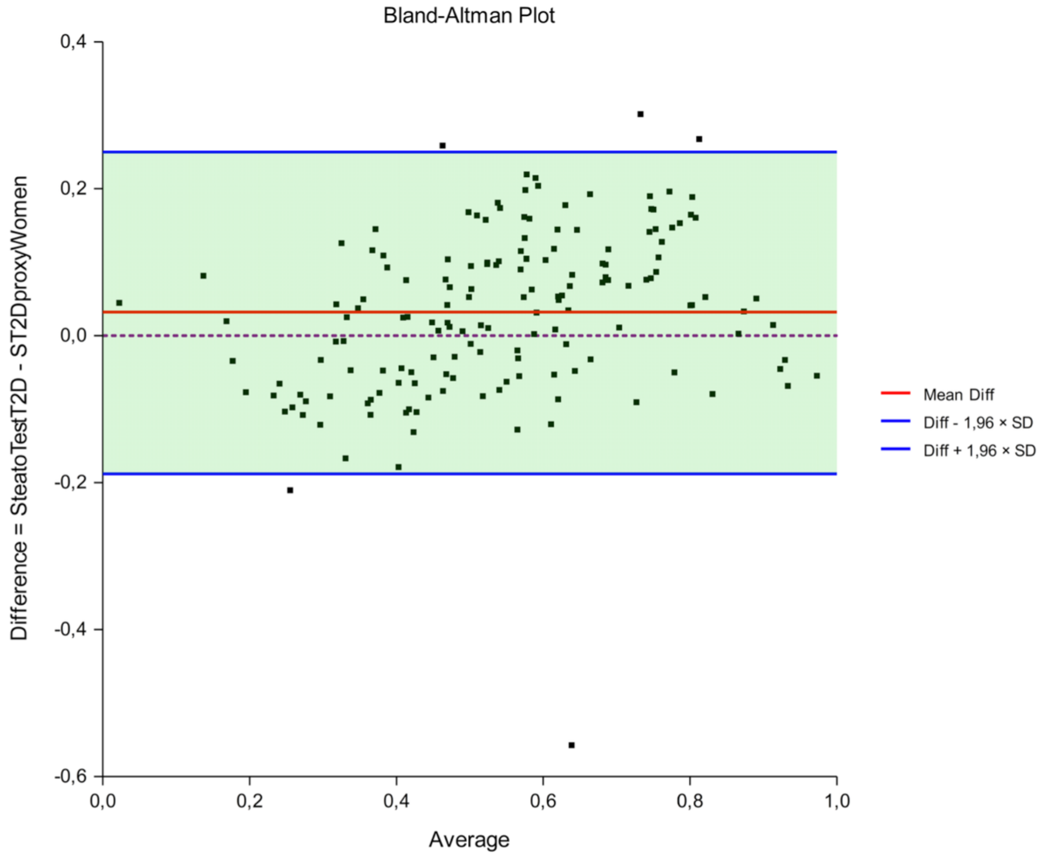

Descriptive Statistics

Variable	Count	Mean	Standard Deviation	95,0% LCL of Mean	95,0% UCL of Mean
SteatoTestT2D	159	0,5608608	0,2093648	0,528067	0,5936547
ST2DproxyWomen	159	0,5294408	0,1746827	0,5020794	0,5568022
Difference	159	0,03142006	0,1114843	0,01395772	0,0488824

Correlation

Correlation Coefficient = 0,846525

Bland-Altman Analysis: Bias and Limits of Agreement for SteatoTestT2D and ST2DproxyWomen

Limits of Agreement = Diff ± 1,96 × (Std Dev of Difference)

Parameter	Count	Value	Standard Deviation	95,0% LCL of Value	95,0% UCL of Value
Bias (Difference)	159	0,03142006	0,1114843	0,01395772	0,0488824
Lower Limit of Agreement	159	-0,1870892	0,01514147	-0,216995	-0,1571834
Upper Limit of Agreement	159	0,2499293	0,01514147	0,2200235	0,2798351

Test of Normality of Differences Assumption

Assumption	Value	Prob Level	Decision (α = 0,050)
Shapiro-Wilk	0,950	0,0000	Reject normality

Evaluation of Assumptions Plots

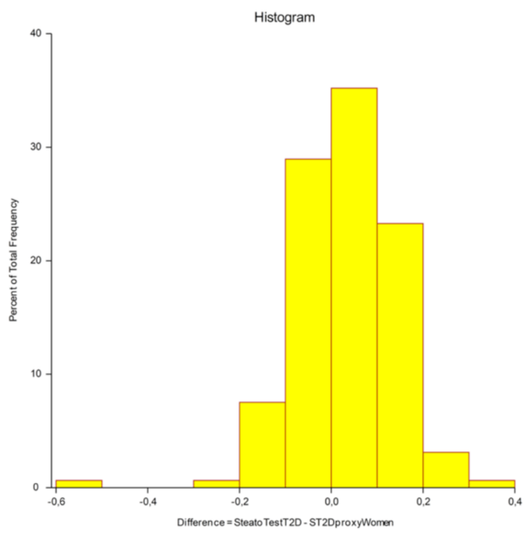

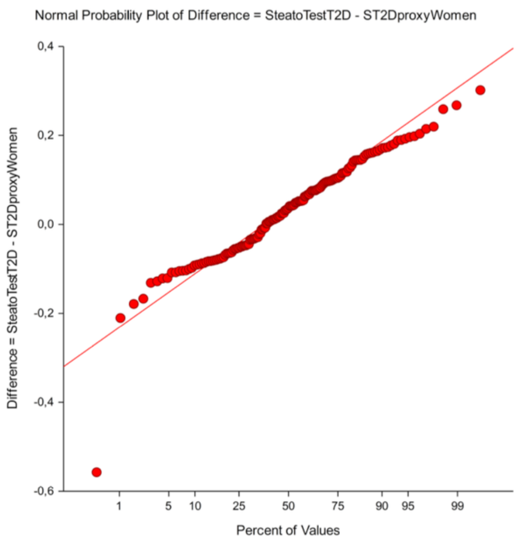

Evaluation of Assumptions Plots (Continued)

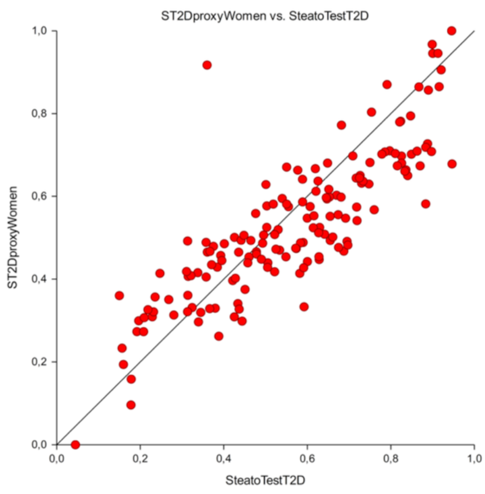

Panel C. Men SteatoTest NITs.

Bland-Altman Plot

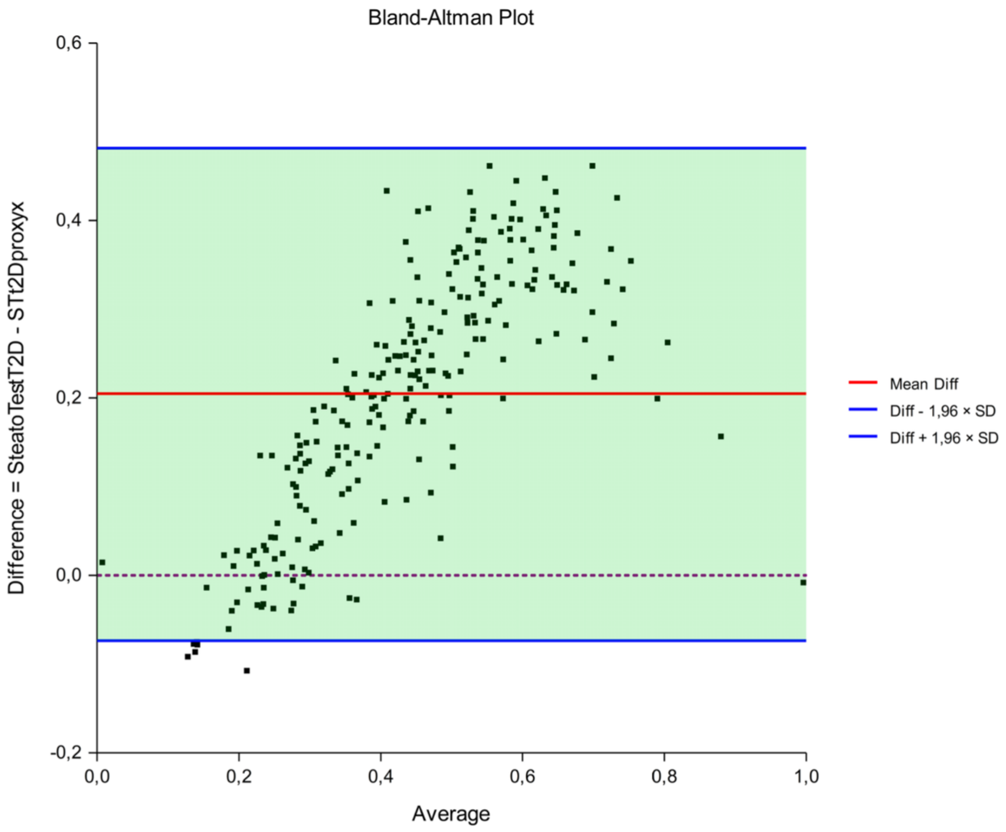

Descriptive Statistics

Variable	Count	Mean	Standard Deviation	95,0% LCL of Mean	95,0% UCL of Mean
SteatoTestT2D	243	0,5384889	0,2187866	0,5108422	0,5661356
STt2Dproxyx	243	0,3344615	0,1144226	0,3200026	0,3489203
Difference	243	0,2040274	0,1416782	0,1861244	0,2219304

Correlation

Correlation Coefficient = 0,816633

Bland-Altman Analysis: Bias and Limits of Agreement for SteatoTestT2D and STt2Dproxyx

Limits of Agreement = Diff ± 1,96 × (Std Dev of Difference)

Parameter	Count	Value	Standard Deviation	95,0% LCL of Value	95,0% UCL of Value
Bias (Difference)	243	0,2040274	0,1416782	0,1861244	0,2219304
Lower Limit of Agreement	243	-0,07366176	0,01555392	-0,1043001	-0,0430234
Upper Limit of Agreement	243	0,4817166	0,01555392	0,4510783	0,512355

Test of Normality of Differences Assumption

Assumption	Value	Prob Level	Decision (α = 0,050)
Shapiro-Wilk	0,969	0,0000	Reject normality

Evaluation of Assumptions Plots

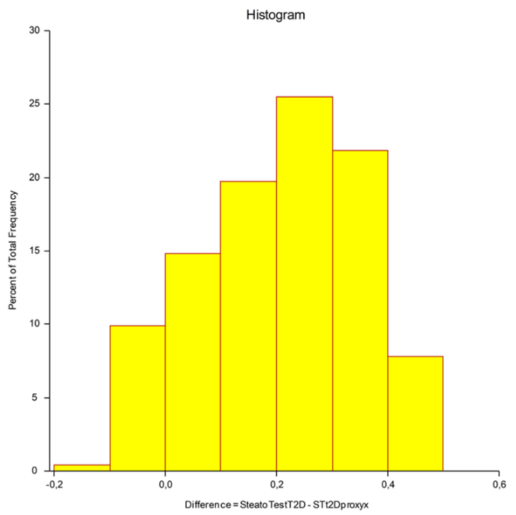

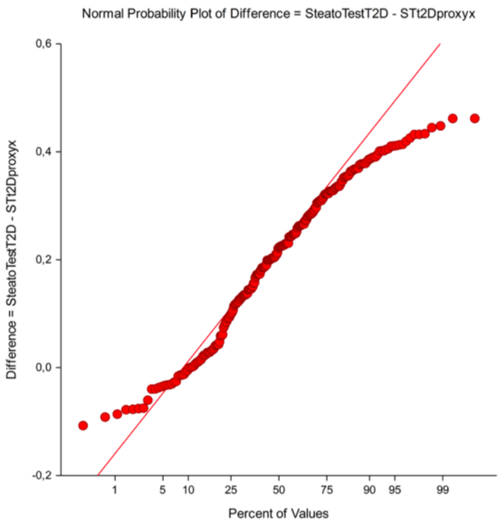

Evaluation of Assumptions Plots (Continued)

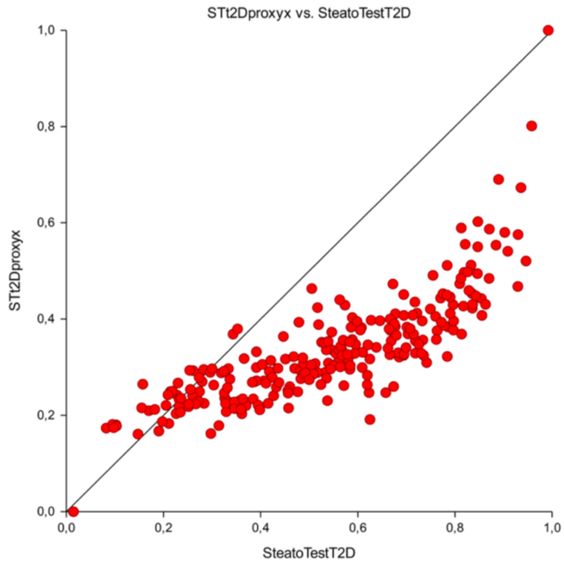

Panel D. Women Nash NITs.

Bland-Altman Plot

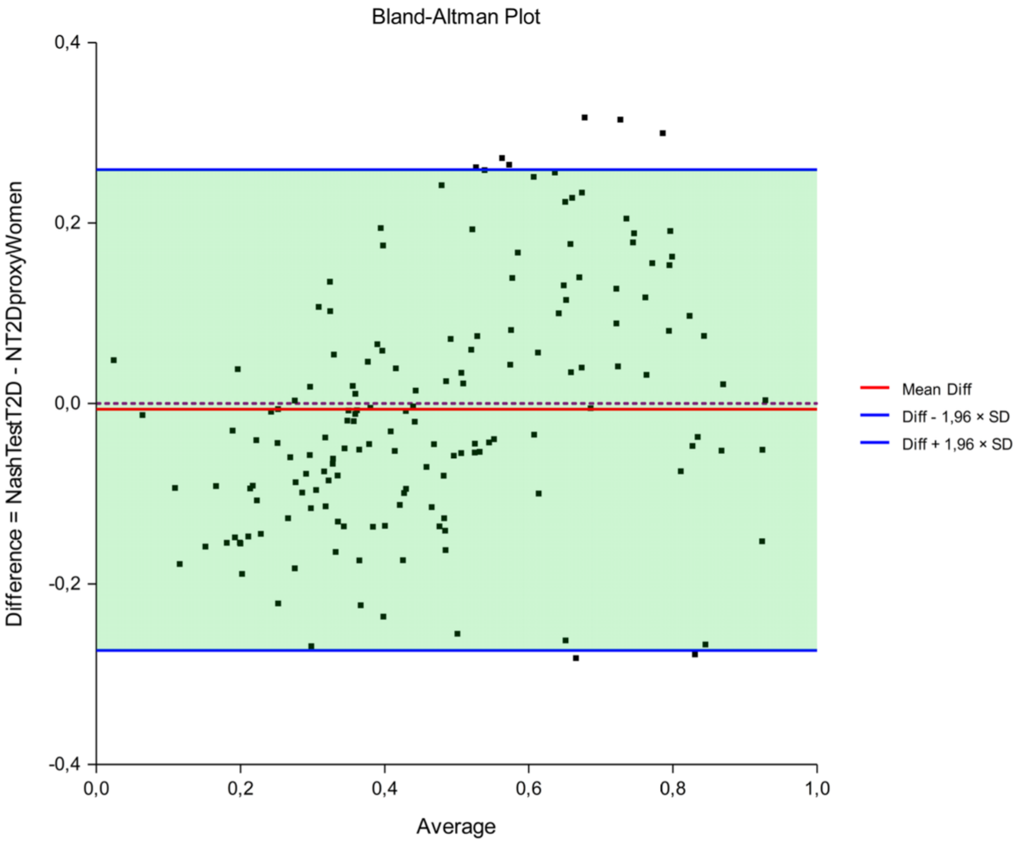

Test

Descriptive Statistics

Variable	Count	Mean	Standard Deviation	95,0% LCL of Mean	95,0% UCL of Mean
NashTestT2D	159	0,4708581	0,241251	0,4330698	0,5086465
NT2DproxyWomen	159	0,4775018	0,1892405	0,4478602	0,5071435
Difference	159	-0,006643695	0,1358088	-0,0279161	0,01462871

Correlation

Correlation Coefficient = 0,827630

Bland-Altman Analysis: Bias and Limits of Agreement for NashTestT2D and NT2DproxyWomen

Limits of Agreement = Diff ± 1,96 × (Std Dev of Difference)

Parameter	Count	Value	Standard Deviation	95,0% LCL of Value	95,0% UCL of Value
Bias (Difference)	159	-0,006643695	0,1358088	-0,0279161	0,01462871
Lower Limit of Agreement	159	-0,2728288	0,01844514	-0,3092597	-0,236398
Upper Limit of Agreement	159	0,2595415	0,01844514	0,2231106	0,2959723

Test of Normality of Differences Assumption

Assumption	Value	Prob Level	Decision (α = 0,050)
Shapiro-Wilk	0,978	0,0136	Reject normality

Evaluation of Assumptions Plots

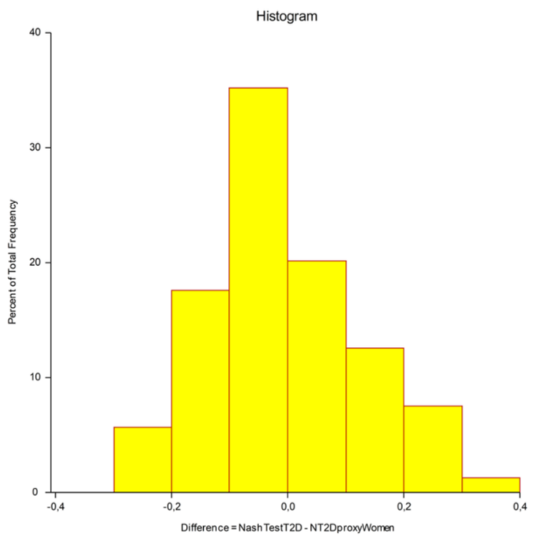

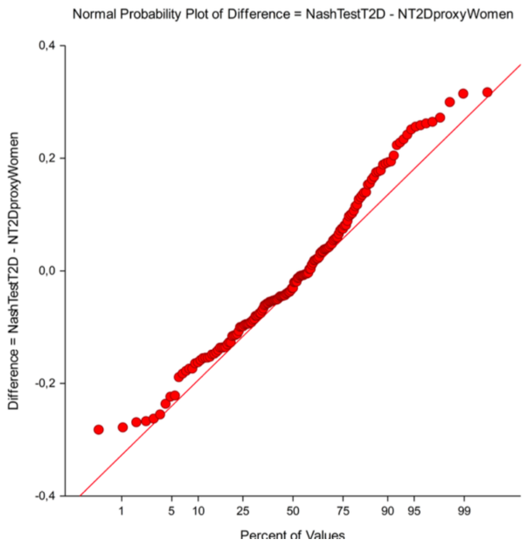

Evaluation of Assumptions Plots (Continued)

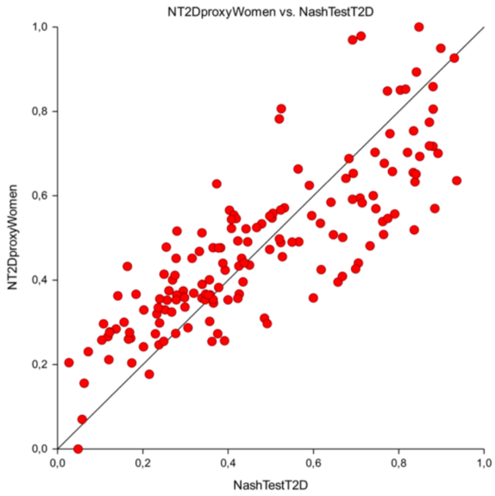

Panel E. Men Nash NITs.

Bland-Altman Plot

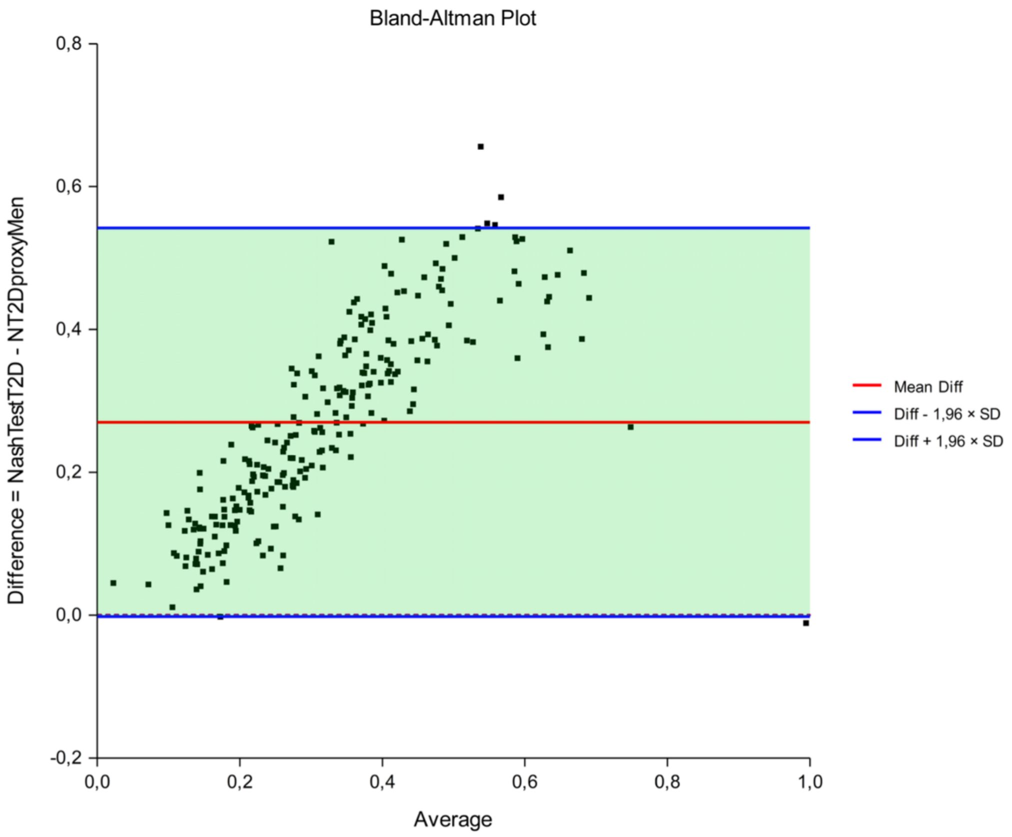

Descriptive Statistics

Variable	Count	Mean	Standard Deviation	95,0% LCL of Mean	95,0% UCL of Mean
NashTestT2D	243	0,45756	0,2066174	0,431451	0,4836689
NT2DproxyMen	243	0,1884001	0,1036447	0,1753032	0,201497
Difference	243	0,2691599	0,1388053	0,2516199	0,2866998

Correlation

Correlation Coefficient = 0,797721

Bland-Altman Analysis: Bias and Limits of Agreement for NashTestT2D and NT2DproxyMen

Limits of Agreement = Diff ± 1,96 × (Std Dev of Difference)

Parameter	Count	Value	Standard Deviation	95,0% LCL of Value	95,0% UCL of Value
Bias (Difference)	243	0,2691599	0,1388053	0,2516199	0,2866998
Lower Limit of Agreement	243	-0,002898489	0,01523853	-0,03291558	0,0271186
Upper Limit of Agreement	243	0,5412183	0,01523853	0,5112011	0,5712354

Test of Normality of Differences Assumption

Assumption	Value	Prob Level	Decision (α = 0,050)
Shapiro-Wilk	0,978	0,0007	Reject normality

Evaluation of Assumptions Plots

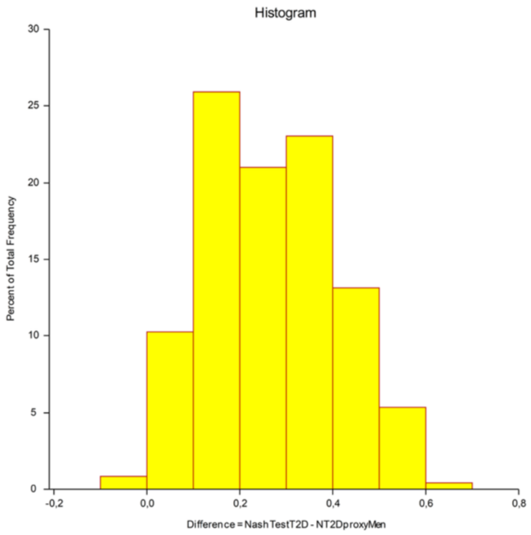

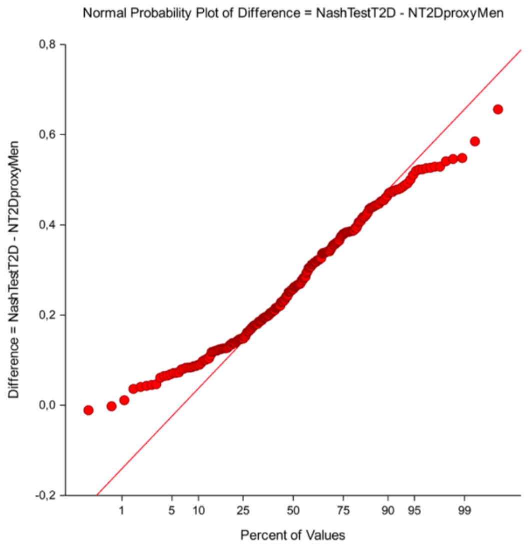

Evaluation of Assumptions Plots (Continued)

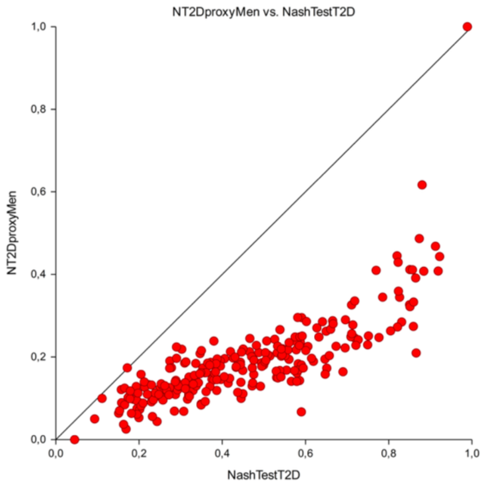

Panel F. Women Fibrosis NITs.

Bland-Altman Plot

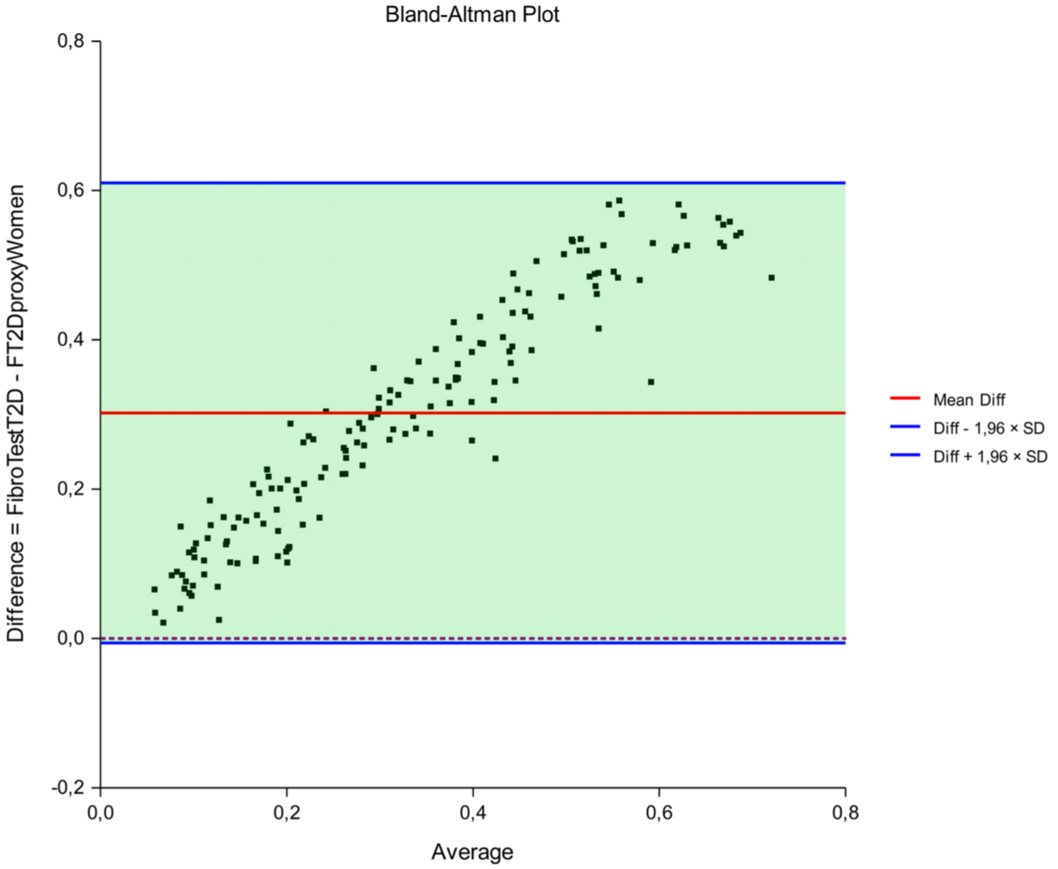

Descriptive Statistics

Variable	Count	Mean	Standard Deviation	95,0% LCL of Mean	95,0% UCL of Mean
FibroTestT2D	159	0,4794624	0,2505656	0,4402151	0,5187098
FT2DproxyWomen	159	0,177919	0,102992	0,1617868	0,1940511
Difference	159	0,3015434	0,157235	0,2769149	0,3261719

Correlation

Correlation Coefficient = 0,942943

Bland-Altman Analysis: Bias and Limits of Agreement for FibroTestT2D and FT2DproxyWomen

Limits of Agreement = Diff ± 1,96 × (Std Dev of Difference)

Parameter	Count	Value	Standard Deviation	95,0% LCL of Value	95,0% UCL of Value
Bias (Difference)	159	0,3015434	0,157235	0,2769149	0,3261719
Lower Limit of Agreement	159	-0,006637138	0,02135519	-0,04881561	0,03554133
Upper Limit of Agreement	159	0,609724	0,02135519	0,5675455	0,6519025

Test of Normality of Differences Assumption

Assumption	Value	Prob Level	Decision (α = 0,050)
Shapiro-Wilk	0,957	0,0001	Reject normality

Evaluation of Assumptions Plots

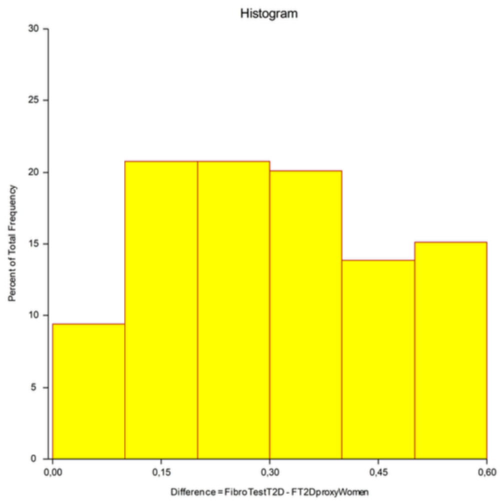

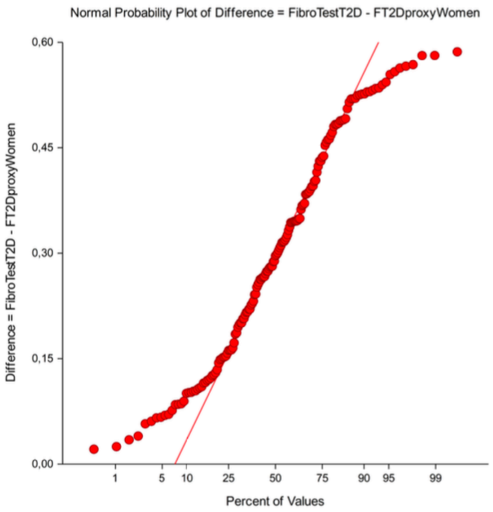

Evaluation of Assumptions Plots (Continued)

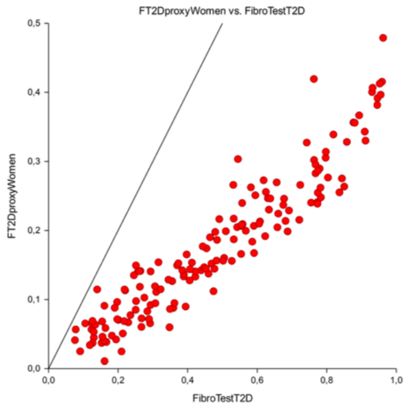

Panel G. Men Fibrosis NITs.

Bland-Altman Plot

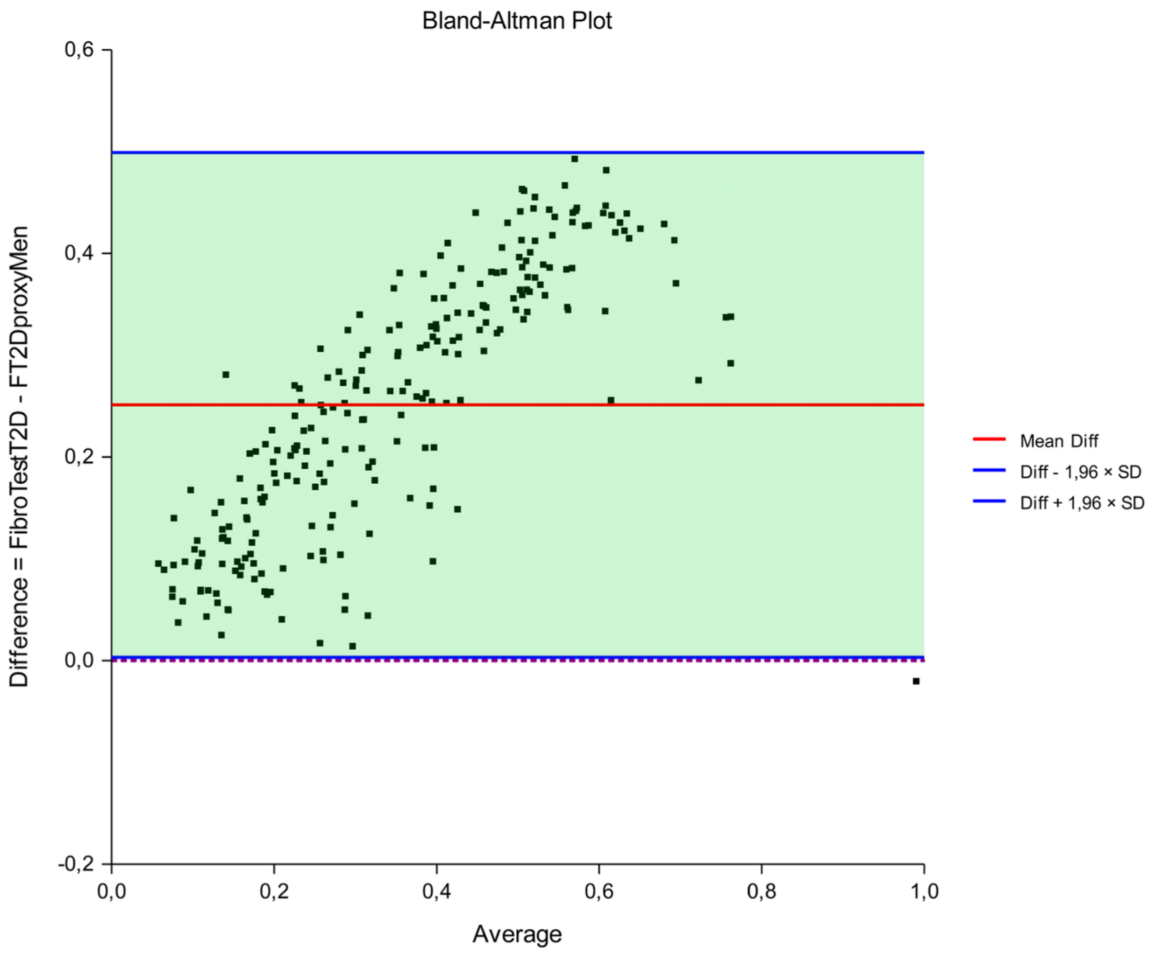

Descriptive Statistics

Variable	Count	Mean	Standard Deviation	95,0% LCL of Mean	95,0% UCL of Mean
FibroTestT2D	243	0,4731796	0,2254781	0,4446873	0,5016718
FT2DproxyMen	243	0,2216423	0,129835	0,2052359	0,2380487
Difference	243	0,2515373	0,1265051	0,2355516	0,2675229

Correlation

Correlation Coefficient = 0,882905

Bland-Altman Analysis: Bias and Limits of Agreement for FibroTestT2D and FT2DproxyMen

Limits of Agreement = Diff ± 1,96 × (Std Dev of Difference)

Parameter	Count	Value	Standard Deviation	95,0% LCL of Value	95,0% UCL of Value
Bias (Difference)	243	0,2515373	0,1265051	0,2355516	0,2675229
Lower Limit of Agreement	243	0,003587287	0,01388817	-0,02376984	0,03094442
Upper Limit of Agreement	243	0,4994873	0,01388817	0,4721301	0,5268444

Test of Normality of Differences Assumption

Assumption	Value	Prob Level	Decision (α = 0,050)
Shapiro-Wilk	0,960	0,0000	Reject normality

Evaluation of Assumptions Plots

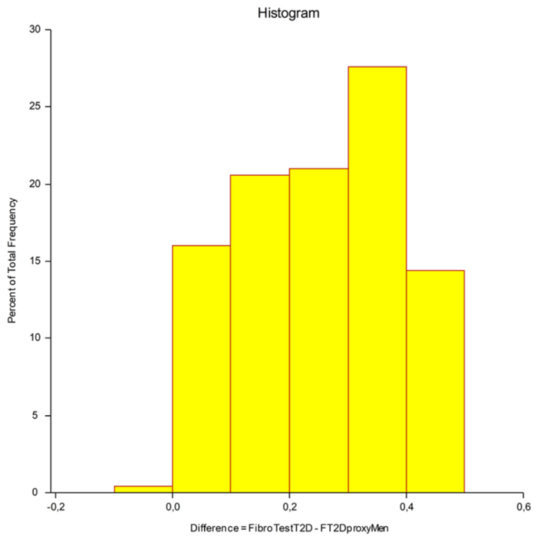

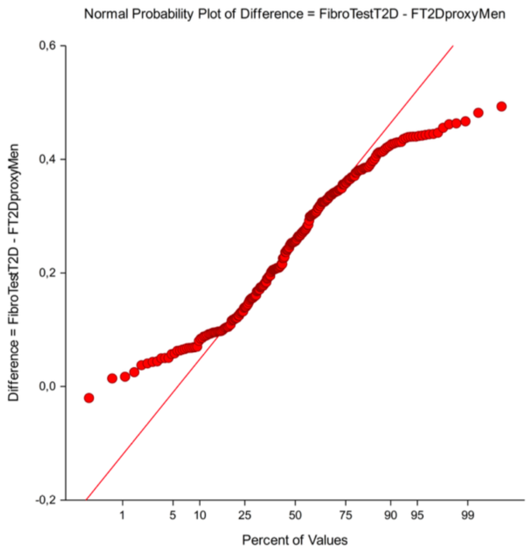

Evaluation of Assumptions Plots (Continued)

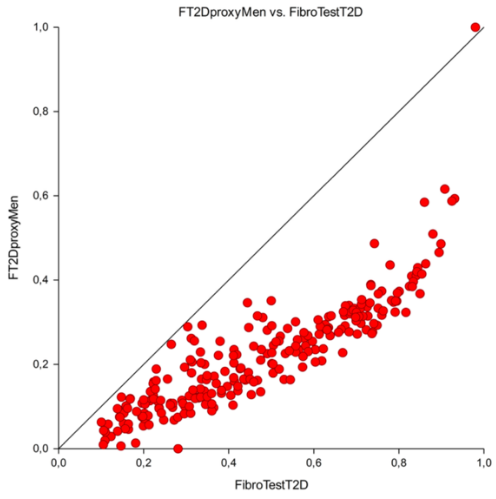

-Supplementary-File-S7. Sensitivity Analyses

bAUROCs of NashTest-T2D vs AST for activity in BARICAN participants

Panel A. First biopsy for activity at surgery,

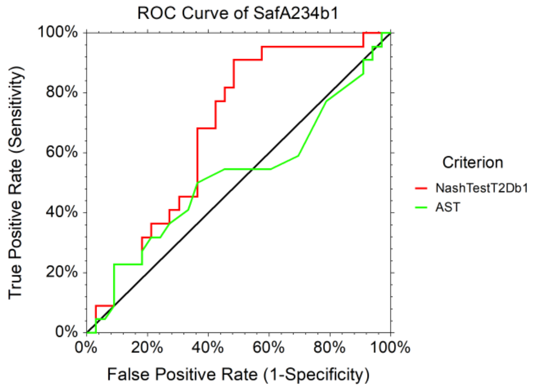

Panel B. Second biopsy for activity after surgery, MashTest-T2D vs AST

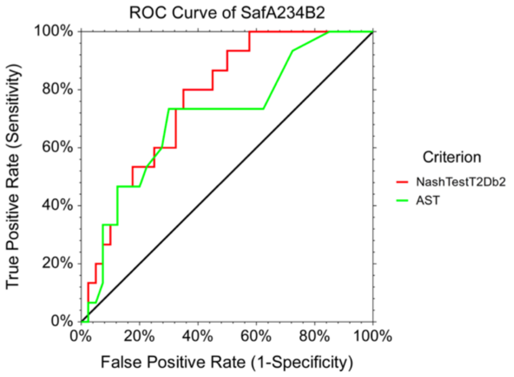

Panel C. Second biopsy after surgery for steatosis, SteatoTest-T2D vs triglycerides.

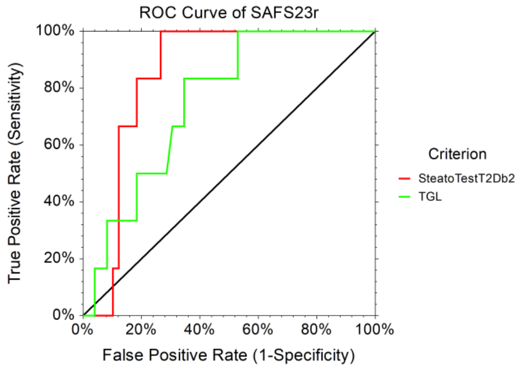

Supplementary-File-S8. Updated meta-analysis overview.

History of the four most cited publications of circulating biomarkers combinations

The first publications of the four most cited circulating biomarkers combinations for MASLD were initially published in patients with chronic hepatitis C or mixed causes between 2001 to 2005:

1. Imbert-Bismut F, Ratziu V, Pieroni L, et al. Biochemical markers of liver fibrosis in patients with hepatitis C virus infection: a prospective study. *Lancet*. 2001;357(9262):1069-1075. doi:10.1016/S0140-6736(00)04258-6
2. Rosenberg WM, Voelker M, Thiel R, et al. Serum markers detect the presence of liver fibrosis: a cohort study. *Gastroenterology*. 2004;127(6):1704-1713. doi:10.1053/j.gastro.2004.08.052
3. Adams LA, Bulsara M, Rossi E, et al. Hepascore: an accurate validated predictor of liver fibrosis in chronic hepatitis C infection. *Clin Chem*. 2005;51(10):1867-1873. doi:10.1373/clinchem.2005.048389
4. Calès P, Oberti F, Michalak S, et al. A novel panel of blood markers to assess the degree of liver fibrosis. *Hepatology*. 2005;42(6):1373-1381. doi:10.1002/hep.20935

Two markers, FibroTest and Fibrometer have an optimized version for MASLD: FibroTest-T2D and FibroMeter-V2G respectively.

History of the 15 publications (22 comparisons) of the four most cited circulating biomarkers combinations for MASLD between 2006 to 2024

One study compared four markers: FibroTest, Hepascore, Fibrometer-NAFLD and Fibrometer-V2G, 4 comparisons.

1. Boursier J, Vergniol J, Guillet A, et al. Diagnostic accuracy and prognostic significance of blood fibrosis tests and liver stiffness measurement by FibroScan in non-alcoholic fatty liver disease. *J Hepatol*. 2016;65(3):570-578. doi:10.1016/j.jhep.2016.04.023

Two studies compared three markers: FibroTest, FT-T2D, and Fibrometer-V2G, 6 comparisons.

1. Ratziu V, Massard J, Charlotte F, et al. Diagnostic value of biochemical markers (FibroTest-FibroSURE) for the prediction of liver fibrosis in patients with non-alcoholic fatty liver disease. *BMC Gastroenterol*. 2006;6:6. Published 2006 Feb 14. doi:10.1186/1471-230X-6-652.
2. Guillaume M, Moal V, Delabaudiere C, et al. Direct comparison of the specialised blood fibrosis tests FibroMeter^{V2G} and Enhanced Liver Fibrosis score in patients with non-alcoholic fatty liver disease from tertiary care centres. *Aliment Pharmacol Ther*. 2019;50(11-12):1214-1222. doi:10.1111/apt.15529

Two studies compared same markers (Fibrometer-Nafld vs Fibrometer-V2G with same patients (not all) in both studies.

For the F3F4 diagnosis, the authors concluded that accuracy was moderate for FibroTest with a bAUROC=0.78, good for ELF with a bAUROC=0.87, and good for FibroMeter with a bAUROC=0.84; for Hepascore, not enough studies were available

For the F2F3F4 diagnosis, they concluded that accuracy was good for FibroTest with a bAUROC=0.86 and good for FibroMeter with a bAUROC=0.88; for ELF and Hepascore, not enough studies were available. For Hepascore, which also included A2M, they concluded that

not enough studies were available to perform a meta-analysis of accuracy. They stated that the ELF score was not accessible to frontline health professionals, which may represent a barrier to the detection of liver fibrosis.

The authors were surprised by the lower bAUROCs of the FibroMeter-NAFLD reconstructed for this context of use in comparison to the previous versions of FibroMeter-v2G constructed mostly in viral hepatitis. The authors speculated that this difference was due to the inclusion of A2M, a direct marker of liver fibrosis, in a non-invasive test, improving its diagnostic accuracy. We agree with this comment as we described 30 years ago in 525 patients with alcoholic liver disease that A2M, often considered a marker of inflammation, was indeed associated with fibrosis stages independently of apoA1 and GGT.

How did our results improve this overview?

Among the 22 comparisons, nine concerned Fibrotest or FT-T2D, six FibroMeter-NAFLD or FibroMeter-v2G, four ELF, and three Hepascore. Less than 50% of studies used the recommended wAUROCs. In the absence of such methods, we used the adjusted bAUROC assessed using an adjusted DANA-Index (**Supplementary-FileS3**). Eight studies used head-to-head comparisons, which prevented spectrum bias and reduced the impact of CFs (**Table 3**).

Sixteen studies (76%) gave the biopsy median length, but only two studies used it to stratify the AUROCs (FibroTest). As expected, no median length >30 mm was identified, and only two recent studies (FibroTest) used the EPOS 7-tier score.

These results confirmed the risks of ranking circulating fibrosis markers on the meta-analyses of bAUROCs without accounting for the inappropriate sensitivity of the 5-tier score, the spectrum bias, and the length of the biopsy. If a bAUROC ≥0.80 was applied as a selection criterion (milestone),[63,64,65] 12 comparisons reached this cutoff (57%): FibroTest (n=4), FT-T2D (n=0), ELF (n=2), Hepascore (n=2), FibroMeter-NAFLD (n=2), and FibroMeter-v2G (n=2). However, a simple adjustment by the DANA index, which considers the spectrum bias, reduced this milestone selection to only six markers of interest (27%): FibroTest (n=3), FibroMeter-NAFLD (n=1), FibroMeter-v2G (n=1), ELF (n=1), and Hepascore (n=1); see **Table 3**.

Supplementary-File-S9. References of the 9 studies of Table 3 (Total 18 studies) not detailed in the article.

Nine studies of Table 3 not detailed in the article.

- Adams LA, George J, Bugianesi E, et al. Complex non-invasive fibrosis models are more accurate than simple models in non-alcoholic fatty liver disease. *J Gastroenterol Hepatol*. 2011;26(10):1536-1543. doi:10.1111/j.1440-1746.2011.06774.x49.
- Aykut UE, Akyuz U, Yesil A, et al. A comparison of FibroMeter™ NAFLD Score, NAFLD fibrosis score, and transient elastography as noninvasive diagnostic tools for hepatic fibrosis in patients with biopsy-proven non-alcoholic fatty liver disease. *Scand J Gastroenterol*. 2014;49(11):1343-1348. doi:10.3109/00365521.2014.958099
- Anstee QM, Lawitz EJ, Alkhouri N, et al. Noninvasive Tests Accurately Identify Advanced Fibrosis due to NASH: Baseline Data From the STELLAR Trials. *Hepatology*. 2019;70(5):1521-1530. doi:10.1002/hep.30842
- Boursier J, Vergniol J, Guillet A, et al. Diagnostic accuracy and prognostic significance of blood fibrosis tests and liver stiffness measurement by FibroScan in non-alcoholic fatty liver disease. *J Hepatol*. 2016;65(3):570-578. doi:10.1016/j.jhep.2016.04.023
- Bril F, McPhaul MJ, Caulfield MP, et al. Performance of the SteatoTest, ActiTest, NashTest and FibroTest in a multiethnic cohort of patients with type 2 diabetes mellitus. *J Investig Med*. 2019;67(2):303-311. doi:10.1136/jim-2018-000864
- Calès P, Lainé F, Boursier J, et al. Comparison of blood tests for liver fibrosis specific or not to NAFLD. *J Hepatol*. 2009;50(1):165-173. doi:10.1016/j.jhep.2008.07.035Nine
- Guillaume M, Moal V, Delabaudiere C, et al. Direct comparison of the specialised blood fibrosis tests FibroMeter^{V2G} and Enhanced Liver Fibrosis score in patients with non-alcoholic fatty liver disease from tertiary care centres. *Aliment Pharmacol Ther*. 2019;50(11-12):1214-1222. doi:10.1111/apt.15529
- Miele L, De Michele T, Marrone G, et al. Enhanced liver fibrosis test as a reliable tool for assessing fibrosis in nonalcoholic fatty liver disease in a clinical setting. *Int J Biol Markers*. 2017;32(4):e397-e402. Published 2017 Oct 31. doi:10.5301/ijbm.5000292
- Subasi CF, Aykut UE, Yilmaz Y. Comparison of noninvasive scores for the detection of advanced fibrosis in patients with nonalcoholic fatty liver disease. *Eur J Gastroenterol Hepatol*. 2015;27(2):137-141. doi:10.1097/MEG.0000000000000255

**22 comparisons in 18 published studies**	
Author Year	**Reference**
**FibroTest**	
Ratziu 2006[39] first	Ratziu V, Massard J, Charlotte F, et al. Diagnostic value of biochemical markers (FibroTest-FibroSURE) for the prediction of liver fibrosis in patients with non-alcoholic fatty liver disease. *BMC Gastroenterol.* 2006;6:6.
Ratziu 2006[39] validation	
Lassailly 2011[34]	Lassailly G, Caiazzo R, Hollebecque A, et al. Validation of noninvasive biomarkers (FibroTest, SteatoTest, and NashTest) for prediction of liver injury in patients with morbid obesity. *Eur J Gastroenterol Hepatol.* 2011;23(6):499-506.
**Adams 2011[*]**	**Adams LA, George J, Bugianesi E, et al. Complex non-invasive fibrosis models are more accurate than simple models in non-alcoholic fatty liver disease. *J Gastroenterol Hepatol.* 2011;26(10):1536-1543.**
Munteanu 2016[49]	Munteanu M, Tiniakos D, Anstee Q, et al. Diagnostic performance of FibroTest, SteatoTest and ActiTest in patients with NAFLD using the SAF score as histological reference. *Aliment Pharmacol Ther.* 2016;44(8):877-889.
Boursier 2016[^,w]	Boursier J, Vergniol J, Guillet A, et al. Diagnostic accuracy and prognostic significance of blood fibrosis tests and liver stiffness measurement by FibroScan in non-alcoholic fatty liver disease. *J Hepatol.* 2016;65(3):570-578.
**Bril 2019[*]**	**Bril F, McPhaul MJ, Caulfield MP, et al. Performance of the SteatoTest, ActiTest, NashTest and FibroTest in a multiethnic cohort of patients with type 2 diabetes mellitus. *J Investig Med.* 2019;67(2):303-311.**
Poynard 2023[21,^,i,°,b,l,r,w]	Poynard T, Paradis V, Mullaert J, et al. Prospective external validation of a new non-invasive test for the diagnosis of non-alcoholic steatohepatitis in patients with type 2 diabetes. *Aliment Pharmacol Ther.* 2021;54(7):952-966.
**FT-T2D**	
Poynard 2023[21,^,i,°,b,l,r,w]	
**ELF**	
Miele 2017[*]	Miele L, De Michele T, Marrone G, et al. Enhanced liver fibrosis test as a reliable tool for assessing fibrosis in nonalcoholic fatty liver disease in a clinical setting. *Int J Biol Markers.* 2017;32(4):e397-e402.
**Anstee 2019[*]**	**Anstee QM, Lawitz EJ, Alkhouri N, et al. Noninvasive Tests Accurately Identify Advanced Fibrosis due to NASH: Baseline Data From the STELLAR Trials. *Hepatology.* 2019;70(5):1521-1530.**
Guillaume 2019[^,w,*]	Guillaume M, Moal V, Delabaudiere C, et al. Direct comparison of the specialised blood fibrosis tests FibroMeter^{V2G} and Enhanced Liver Fibrosis score in patients with non-alcoholic fatty liver disease from tertiary care centres. *Aliment Pharmacol Ther.* 2019;50(11-12):1214-1222.
Arai 2024[r]	Arai T, Takahashi H, Seko Y, et al. Accuracy of the Enhanced Liver Fibrosis Test in Patients With Type 2 Diabetes Mellitus and Its Clinical Implications. *Clin Gastroenterol Hepatol.* 2024;22(4):789-797
**Hepascore**	
Adams 2011[^]	Adams LA, George J, Bugianesi E, et al. Complex non-invasive fibrosis models are more accurate than simple models in non-alcoholic fatty liver disease. *J Gastroenterol Hepatol.* 2011;26(10):1536-1543.
Bertot 2023[38,r]	Bertot LC, Jeffrey GP, de Boer B, et al. Comparative Accuracy of Clinical Fibrosis Markers, Hepascore and Fibroscan® to Detect Advanced Fibrosis in Patients with Nonalcoholic Fatty Liver Disease. *Dig Dis Sci.* 2023;68(6):2757-2767.
**Boursier 2016[^,w]**	**Boursier J, Vergniol J, Guillet A, et al. Diagnostic accuracy and prognostic significance of blood fibrosis tests and liver stiffness measurement by FibroScan in non-alcoholic fatty liver disease. *J Hepatol.* 2016;65(3):570-578.**
**FibroMeter NAFLD**	
**Cales 2009[*]**	**Calès P, Lainé F, Boursier J, et al. Comparison of blood tests for liver fibrosis specific or not to NAFLD. *J Hepatol.* 2009;50(1):165-173.**
**Aykut 2014[t,*]**	**Aykut UE, Akyuz U, Yesil A, et al. A comparison of FibroMeter™ NAFLD Score, NAFLD fibrosis score, and transient elastography as noninvasive diagnostic tools for hepatic fibrosis in patients with biopsy-proven non-alcoholic fatty liver disease. *Scand J Gastroenterol.* 2014;49(11):1343-1348.**
**Boursier 2016[^,w,*]**	**Boursier J, Vergniol J, Guillet A, et al. Diagnostic accuracy and prognostic significance of blood fibrosis tests and liver stiffness measurement by FibroScan in non-alcoholic fatty liver disease. *J Hepatol.* 2016;65(3):570-578.**
**Subasi 2015[t,*]**	**Subasi CF, Aykut UE, Yilmaz Y. Comparison of noninvasive scores for the detection of advanced fibrosis in patients with nonalcoholic fatty liver disease. *Eur J Gastroenterol Hepatol.* 2015;27(2):137-141.**
**FibroMeter V2G**	
**Boursier 2016[^,w,x]**	**Boursier J, Vergniol J, Guillet A, et al. Diagnostic accuracy and prognostic significance of blood fibrosis tests and liver stiffness measurement by FibroScan in non-alcoholic fatty liver disease. *J Hepatol.* 2016;65(3):570-578.**
**Guillaume 2019[^,w,*]**	**Guillaume M, Moal V, Delabaudiere C, et al. Direct comparison of the specialised blood fibrosis tests FibroMeter^{V2G} and Enhanced Liver Fibrosis score in patients with non-alcoholic fatty liver disease from tertiary care centres. *Aliment Pharmacol Ther.* 2019;50(11-12):1214-1222.**

Bold references are those not detailed in the article due to limited number allowed.

Supplementary-File-S8. Protein variability according to CFs: sex, T2D (glucose ≥7 proxy), and BMI in the France and USA subsets

The fibrosis spectrum in large populations at risk of MASLD has more CRN stages F1–F2 (5-tier) than that in populations with chronic hepatitis C or alcoholic liver disease. This reinforces the need to use wAUROC.
Similar increases in apoA1 at perimenopausal age in women with T2D who were not overweight were observed (**Supplementary Figure S6**) in the France-FibroTest (**Panel A and Panel C**) and the USA-FibroTest (**Panel B and Panel D**) subsets in comparison with those in the UK-BioBank general population (**Figure 4 and Supplementary Figure S6 Panel C and Panel G**).
Regardless of age and the absence (**Supplementary Figure S7 Panel A and Panel B**) or presence of T2D (**Supplementary Figure S7 Panel C and Panel D**), the mean haptoglobin was always higher in women with a BMI greater than 27 kg/m2 in comparison with men.
Mean A2M had a U shape both in women and men (**Supplementary Figure S8 Panel A and Panel B**), but the increase in men started 10 years earlier than in women, especially among men with a BMI >30 kg/m2. In men with T2D, the U shape mostly disappeared (**Supplementary Figure S8 Panel D**).

Supplementary-File-S9. FPR in USA and France populations at risk of MASLD

The prevalence of the stage EPOS-F3, were quite similar between countries: 25.8% vs 27.2%, a 1.4% (95% CI -1.2–0.01; P<.001) and stage eF, 17.9% vs 19.2%, a 1.3% (95% CI -1.7°0.01; P<.001). In contrast, the prevalences of 4-CFs were highly different between these two large populations: 54% vs 29% of women, 22% vs 14% for T2D, and 75% vs 43% for overweight, in the USA and France subsets, respectively (all P<.0001).
In the large population of middle-aged participants of the UK-Biobank, and its prospective design several major CTs difference were expected: the percentage of women was close to 50% (48%), the age was older (59 years), and T2D was much less (7.2%) as only healthy participants were included.
Finally, severe obesity (BMI ≥35kg/m2) was much more frequent in the USA (29.7%), compared to 13.7% in the UK-Biobank and 10.6% in the France subsets (all P<.0001; **Supplementary-Table-S3**).
